# Analysis of trace metal distribution in plants with lab-based microscopic X-ray fluorescence imaging

**DOI:** 10.1186/s13007-020-00621-5

**Published:** 2020-06-08

**Authors:** Ana Mijovilovich, Filis Morina, Syed Nadeem Bokhari, Timo Wolff, Hendrik Küpper

**Affiliations:** 1 Biology Centre of the Czech Academy of Sciences, Department of Plant Biophysics & Biochemistry, Institute of Plant Molecular Biology, Branišovská 1160/31, 370 05 Ceske Budejovice, Czech Republic; 2 Bruker Nano GmbH, Am Studio 2D, 12489 Berlin, Germany; 3 Department of Experimental Plant Biology, University of South Bohemia, Branišovská 1160/31, 370 05 Ceske Budejovice, Czech Republic

**Keywords:** Micro X-ray fluorescence, Zinc, Hyperaccumulator, Micronutrients, Leaf age, Root, *Arabidopsis halleri*, *Noccaea caerulescens*, *Glycine max*, *Capsicum annuum*

## Abstract

**Background:**

Many metals are essential for plants and humans. Knowledge of metal distribution in plant tissues in vivo contributes to the understanding of physiological mechanisms of metal uptake, accumulation and sequestration. For those studies, X-rays are a non-destructive tool, especially suited to study metals in plants.

**Results:**

We present microfluorescence imaging of trace elements in living plants using a customized benchtop X-ray fluorescence machine. The system was optimized by additional detector shielding to minimize stray counts, and by a custom-made measuring chamber to ensure sample integrity. Protocols of data recording and analysis were optimised to minimise artefacts. We show that Zn distribution maps of whole leaves in high resolution are easily attainable in the hyperaccumulator *Noccaea caerulescens*. The sensitivity of the method was further shown by analysis of micro- (Cu, Ni, Fe, Zn) and macronutrients (Ca, K) in non-hyperaccumulating crop plants (soybean roots and pepper leaves), which could be obtained in high resolution for scan areas of several millimetres. This allows to study trace metal distribution in shoots and roots with a wide overview of the object, and thus avoids making conclusions based on singular features of tiny spots. The custom-made measuring chamber with continuous humidity and air supply coupled to devices for imaging chlorophyll fluorescence kinetic measurements enabled direct correlation of element distribution with photosynthesis. Leaf samples remained vital even after 20 h of X-ray measurements. Subtle changes in some of photosynthetic parameters in response to the X-ray radiation are discussed.

**Conclusions:**

We show that using an optimized benchtop machine, with protocols for measurement and quantification tailored for plant analyses, trace metal distribution can be investigated in a reliable manner in intact, living plant leaves and roots. Zinc distribution maps showed higher accumulation in the tips and the veins of young leaves compared to the mesophyll tissue, while in the older leaves the distribution was more homogeneous.

## Background

The transition metals Cu, Fe, Mn, Mo, Ni and Zn are essential micronutrients for plants, needed for proper function and structure of many proteins, redox reactions and regulation of transcription factors (e.g. Zn) (general plant reviews by Marschner [1]; Andresen et al. [2]; photosynthesis-oriented review by Yruela [3]). In addition, micronutrients are involved in plant defence responses against abiotic (extreme weather conditions) and biotic stress (pathogens) [2, 4]. Requirements for micronutrients are species-specific, but also vary in relation to plant age, organ and tissue level. Excessive accumulation of micronutrients, however, is detrimental for plants [5].

Plants have developed different strategies for regulating metal homeostasis, which divide them into three categories: excluders, indicators and hyperaccumulators [6]. Excluders keep a constant concentration in the shoots by restricting uptake and enhancing efflux of excess metals. Indicators accumulate metals in the shoots, commensurate to their concentration in the soils. Hyperaccumulators actively accumulate metals even in metal-deficient soils, and metal content in the shoots may reach up to several percent of dry matter; e.g. 1% is the lower threshold for defining a Zn-hyperaccumulator (review by Leitenmaier and Küpper [7]). The same species might belong to different categories for different metals. This is the case of *Noccaea caerulescens*, which hyperaccumulates Zn and Cd but not Cu [8, 9]. *N. caerulescens* has a different strategy to tolerate excess Cu [9] than Zn and Cd [10].

Besides their essential role in metabolism of all organisms, in hyperaccumulating plants metals are a part of the defence mechanism against herbivores and pathogens [7, 11–15]. In non-accumulator organisms, metals are involved in immunity as well, as it is well-known from animals, but at least for Fe it was also already demonstrated in plants [4].

To be able to understand the role of micronutrients in plant stress response but also in growth and development, differential accumulation and requirements within the same organ, and within different tissues have to be considered (reviews by Zhao et al. [16]; van der Ent et al. [17]). For example, cellular metal distribution in the leaves of *N. caerulescens* has been analysed using energy dispersive X-ray microanalysis (EDXMA) in shock-frozen hydrated samples, showing that most of the Zn is accumulated in large epidermal storage cells [18]. The distribution of Cd in *N.* *caerulescens* has been determined by autoradiography to avoid the interference of K in the L-edge of Cd in X-ray analysis like EDXMA [19]. Cd was found in the whole leaf but mostly concentrated in the edge. An important question is if any redistribution of metals occurs with aging. In *N. caerulescens*, more Zn was found in mature leaves than in young leaves [18, 20]. Investigating Zn metabolism and homeostasis in hyperaccumulators like *N.* *caerulescens* benefits from the ideal signal/noise ratio for the accumulated metal. Nevertheless, not all processes can be studied in hyperaccumulator models so that non-accumulators like crop plants need to be studied as well (reviews by Leitenmaier and Küpper [7]; Andresen et al. [2]; Krämer [21]).

X-ray based imaging methods are more challenging in non-accumulators because of the very low concentrations of the most interesting trace metals such as Fe, Cu, Mn and Zn. With most standard benchtop machines, trace metals in low concentrations are not accessible and so far there have been no data on micronutrient detection in the leaves using such machine. A recent publication demonstrated Zn visualization in a “primed” seed. However, this was achieved only by immersing the seeds in a very high Zn concentration (1000 mg kg^−1^) [22] (all concentrations in parts per million (mg kg^−1^)). Using an earlier version of the machine presented in this work, but with a smaller detector area and unshielded, Ramos et al. [23] obtained unquantified images of elemental distribution in soybean seeds (with Zn content ranging from 10th to 100th of mg kg^−1^). In this case, a single detector with a smaller area (30 mm^2^) was sufficient to obtain structural resolution due to the big size of the seed tissues (in the range of mm).

For synchrotron studies, whole plant organs like entire leaves or roots are gigantic objects requiring unfeasible amount of beamtime. Therefore, synchrotron X-ray studies are restricted to small objects/sections. Benchtop machines open the possibility to study the whole object. Furthermore, studies on living samples at synchrotrons are problematic because synchrotrons lack the infrastructure for conducting sophisticated plant experiments such as long-term metal treatments. In addition, to obtain insight into long-term element re-distributions as a result of ontogenesis, abiotic or biotic stress, it is necessary to perform several measurements. This is not possible at synchrotrons due to the beamtime limitation. On the other hand, lab-based benchtop machines situated in plant research institutes enable analysis of plants from all types of experiments in vivo. Several groups have endeavored in developing benchtop micro-X-ray fluorescence (µXRF), either with self-made set-ups [22, 24] or commercial devices [23]. The main advantage of µXRF to study mineral nutrition in plants over mass spectroscopic methods in that it is non-destructive.

In this work we demonstrate the use of a novel optimized benchtop µXRF system with a custom-designed measuring chamber. It enabled visualization of trace metal distribution in living leaf and root samples. Moreover, the system can be used not only for studying metal-hyperaccumulators but also for non-accumulator crop species grown under environmentally relevant conditions. We discuss the difficulties in measuring samples with topography, the choice of filters, focusing problems and possible artifacts as well as the choice of standards for most accurate quantification, which were compared to inductively coupled plasma mass spectrometry (ICP-MS) data. The application example shows the Zn accumulation pattern along the leaf development of *N. caerulescens*, metal distribution in the leaves of *Capsicum annuum* (pepper) and in the root hairs of *Glycine max* (soybean). The quantification was done with standards that match the tissue thickness. Leaf integrity after 20 h of measurements (up to 600 ms on a single spot) was evaluated by measuring photosynthetic parameters. In our work we show how the improved spatial resolution (twofold) and detection efficiency (with about fourfold more detector area) results in cellular resolution in leaves, and tissue resolution in roots, with a much lower dose reducing the chance of radiation damage. The presented system allows studying metal uptake and distribution in vivo, at trace concentrations, without artifacts caused by sample manipulation. Metal distribution can be directly related to primary photochemical reactions and other physiological processes and plant stress responses, which are important for ecological and agricultural studies.

## Methods

### Plant growth

**Fig. 1:**
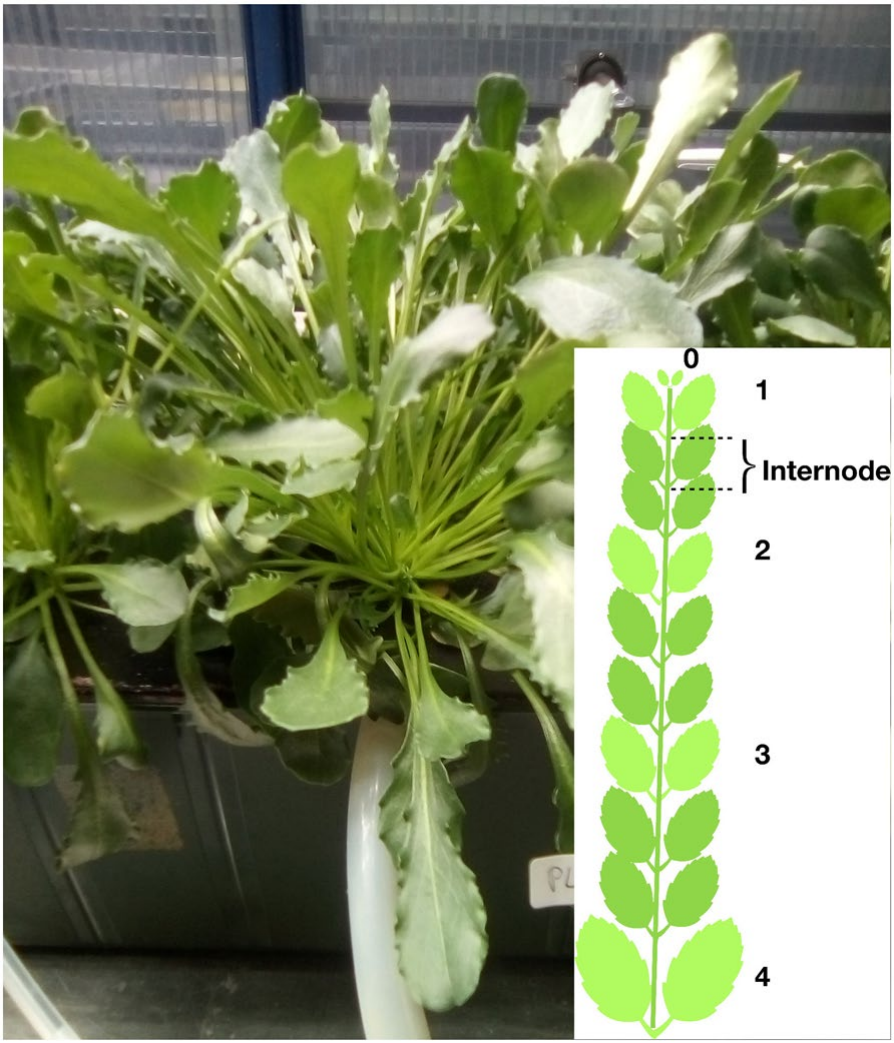
Images of one of the *N.* *caerulescens* plants at the moment of harvest (12 weeks after germination), and the harvesting strategy along one plant shoot branch. Please note that in the scheme the internodes are shown strongly elongated to visualize better the positions of the leaves. The internodes were counted by carefully opening the plant from the top and labelling the leaves. Since in *N.* *caerulescens* the internodes are tiny, it is not possible to see them in the picture

Alpine Penny-cress (*Noccaea caerulescens* (J.Presl & C.Presl) F.K.Mey = formerly *Thlaspi caerulescens* J.Presl & C.Presl), ‘Ganges’ ecotype, was germinated as described earlier [25] and then grown hydroponically for 12 weeks before starting to harvest the leaves for the experiments. The nutrient solution HHNS (“hyperaccumulator hydroponic nutrient solution”) consisted of 1000 μmol L^−1^ Ca(NO_3_)_2_, 500 μmol L^−1^ MgSO_4_, 50 μmol L^−1^ K_2_HPO_4_, 100 μmol L^−1^ KCl, 10 μmol L^−1^ H_3_BO_3_, 0.1 μmol L^−1^ MnSO_4_, 0.2 μmol L^−1^ Na_2_MoO_4_, 0.1 μmol L^−1^ CuSO_4_, 0.5 μmol L^−1^ NiSO_4_, 20 μmol L^−1^ Fe(III)-EDDHA (Fe(III)-ethylenediamine-di(o-hydroxyphenylacetic acid), and 100 μmol L^−1^ ZnSO_4_ as described before [25]. Plants were grown in a greenhouse with automatic ambient light supplementation by a 1:1 mix of cool white and warm white LEDs (Photon System Instruments, Brno, Czech Republic) to achieve a 16 h sinusoidal light cycle with 500 µmol m^−2^ s^−1^ photon flux density. The temperature was regulated to max. 25 °C during the day. Leaves from three plants were sampled from five different points of the rosette covering different stages of development: from the young leaves in the apical meristem rosette down to the elderly, but not senescent, leaves. The harvesting strategy along the rosette is shown in Fig. 1.

Soybean (*Glycine max* (L.) Merr, cultivar ‘Galina’) seeds were germinated on a moistened mixture of perlite and vermiculite (1:3) in the dark at 25 °C to promote radicle development (similarly to e.g. Graham [26]). After 3 days they were transferred to the phytochamber with a sinusoidal light cycle (maximum 500 µmol m^−2^ s^−1^, provided by 8 channels of LEDs to simulate sunlight; Photon System Instruments, Brno, Czech Republic), temperature cycle (25 °C at noon and 18 °C at night) and moisture cycle (40% relative humidity in the afternoon, 60% in the morning before onset of light). Ten days old seedlings (four plants per pot) were transferred to modified ½ strength of the HHNS [10, 25] consisting of 1000 μmol L^−1^ Ca(NO_3_)_2_, 250 μmol L^−1^ MgSO_4_, 250 μmol L^−1^ K_2_HPO_4_,50 μmol L^−1^ KCl, 10 μmol L^−1^ H_3_BO_3_, 0.5 μmol L^−1^ MnSO_4_, 0.2 μmol L^−1^ Na_2_MoO_4_, 0.3 μmol L^−1^ CuSO_4_, 1 μmol L^−1^ ZnSO_4_, 0.5 μmol L^−1^ NiSO_4_ and 20 μmol L^−1^ Fe(III)-EDDHA (Fe(III)-ethylenediamine-di(o-hydroxyphenylacetic acid). The nutrient solution was continuously renewed with a flow rate of 150 mL day^−1^ plant^−1^, which was increased to 250 mL day^−1^ plant^−1^ after 1 week of treatment as described before [25]. After 3 weeks, the second root branch from the top was taken for analysis, and after rinsing in Zn-deficient HHNS the middle part of the root branch was placed in the measuring chamber filled with Zn-HHNS.

Haller’s rockcress (*Arabidopsis halleri* (L.) O’Kane & Al-Shehbaz) plants were grown on soil (mix of 70% commercial peat-free gardening soil and 30% sand) with the same light and temperature conditions as described for *N.* *caerulescens*.

Chili pepper (*Capsicum annuum*, cultivar ‘Kozí Roh’) plants were grown on soil (mix of 70% commercial peat-free gardening soil and 30% sand) under natural daylight and room temperature. Young-mature pepper leaves (just having reached the final size of 3–4 cm) were harvested from approximately 3 to 4 years-old plants.

### Determination of total metal concentration with ICP-MS

For total metal concentration the lyophilized *N.* *caerulescens* and *C.* *annuum* leaves (about 30 mg) were digested in glass tubes with 0.5 mL of a mixture of 85 L 100 L^−1^ of concentrated (70%) HClO_4_ (Suprapur^®^ grade, Carl Roth, Karlsruhe, Germany and 15 L 100 L^−1^ of concentrated (69%) HNO_3_ (Ultrapur^®^ grade, Carl Roth, Karlsruhe, Germany) following the protocol of Zhao et al. [27]. Glycine max roots (four plants per pot) were washed twice with double distilled water (ddH_2_O), tap dried and homogenized in liquid nitrogen. Following lyophilization, about 30 mg of the pooled root samples were used for digestion in three technical replicates.

The glass tubes were uniformly heated using a Fuji PXG4 Thermoblock (AHF Analysentechnik AG, Tübingen, Germany). A program was used for ramping the temperature of the acid mixture to 220 °C for 4 h. The acid mixture was then heated at 220 °C for another hour to dry the digest from acid contents. The digest was then cooled to room temperature and 0.5 mL of 5% HCl (Ultrapur^®^ grade, Carl Roth, Karlsruhe, Germany) was added to each test tube. Afterwards the glass tubes were heated to 90 °C for 1 h to obtain clear solutions. The final volume was made to 1.5 mL with ddH_2_O and stored in 2 mL microcentrifuge vials. Sample solutions were diluted 7000× with 0.2% HNO_3_. Indium was added as internal standard at 1 µg/L to each test solution. The Inductively Coupled Plasma (ICP) multi-element standard solution VI (Merck KGa, Darmstadt, Germany) was used for preparation of several ranges of calibration points. The solution was chosen as standard since the matrix effect was negligible for the quantification of analytes in plant digests. The sector field Inductively Coupled Plasma Mass Spectrometry (ICP-sfMS) Element XR-2 with jet interface (Thermo Fisher Scientific, Bremen, Germany) was used for the elemental analysis of the plant digests. The instrument was optimally tuned to reduce the potential interferences by choosing low, medium and high resolutions (similar to Andresen et al., 2013  [28]). Oxide formation rate was acceptably low as monitored by CeO^+^/Ce^+^. The typical operating conditions of the ICP-sfMS were: RF power: 1250 W, spray chamber temperature: 2 °C, oxide ratio CeO^+^/Ce^+^: 1.0–1.2%, Doubly charged Ce^2+^/Ce^+^: 1.0–1.2%, auxiliary Gas: 0.8 L min^−1^, Sample gas flow: 1.20 L min^−1^ (variable), Cool gas: 16 L min^−1^, Extraction lenses: − 2000 V, Low resolution: 300, Medium resolution: 4000, High resolution: 10,000, Interface cones: Ni sample and H-skimmer cones. In order to avoid contaminations, the ICP-MS is located in a cleanroom with air filtration. All measurements follow a “metal free” protocol, proved in earlier works involving metal deficiency [29], which forbids metal tools and is using Polytetrafluoroethylene (PTFE) tools and vials made from PFA (Perfluoroalkoxy alkanes; Savillex, Eden Prairie, MN, USA) because of their low metal contamination.

### Mounting of intact samples (leaves and roots) for in vivo measurements

**Fig. 2:**
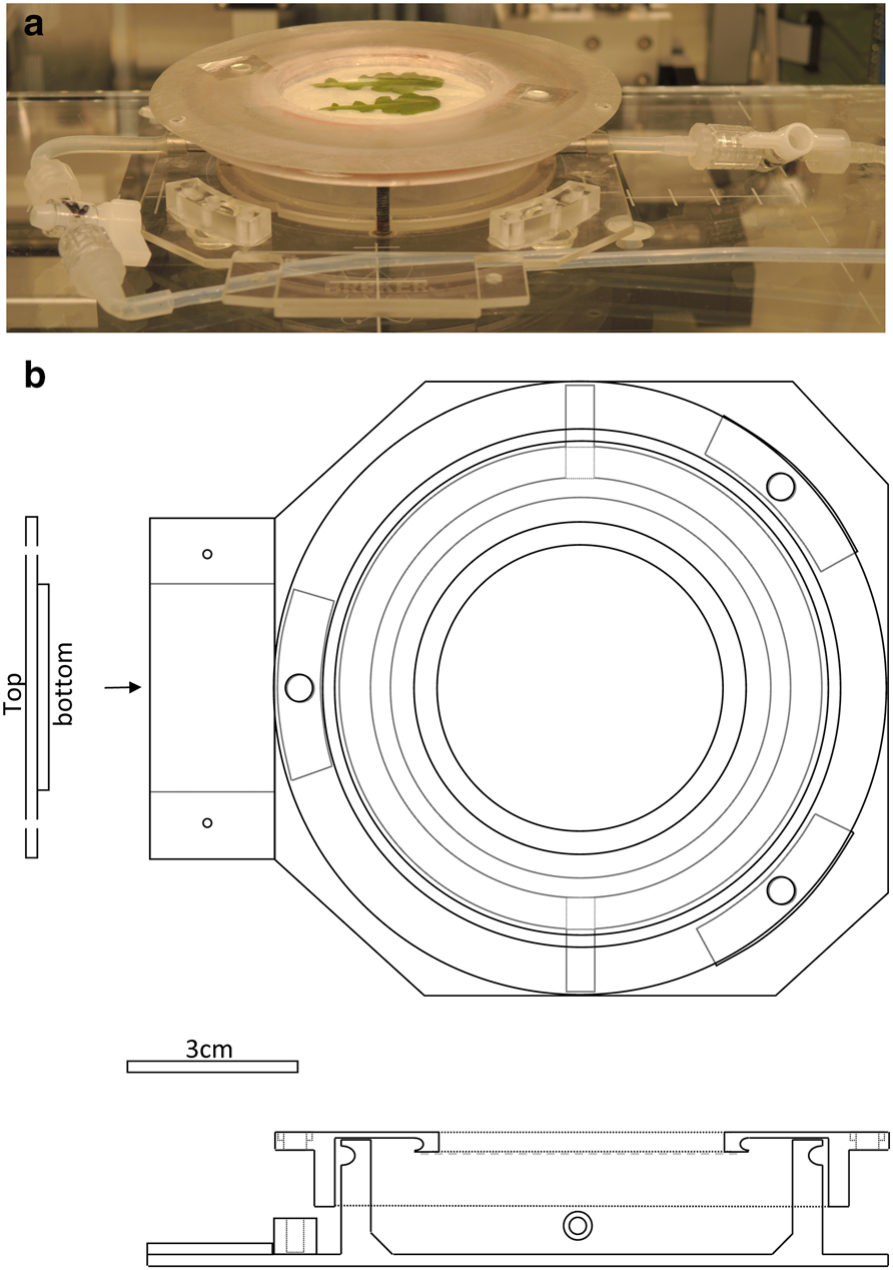
Measuring chamber used for the µXRF and chlorophyll fluorescence kinetic measurements of living samples. **a** Photo, **b** drawing

For all in vivo measurements (chlorophyll fluorescence kinetics and µXRF), samples were mounted in a modified version of the measuring chamber that was originally developed for fluorescence kinetic measurements [25]. The new version of the chamber is shown in Fig. 2. Compared to the previously published version of the chamber, the distance from the sample to the highest point of the chamber had to be drastically reduced because of the short working distance of the benchtop system; when in focus it leaves about 6 mm space between the crash protection plate and the surface of the sample. Therefore, the inlets and outlets for air/nutrient media of the chamber had to be moved from the top to the side, the lid had to be made thinner, and the toric seal (O-ring) holding the leaf assembly had to be moved directly to the lower side of the lid. Finally, the glass window was replaced by a window made of 80 µm thick laser printer foil to diminish the absorption of X-rays.

**Fig. 3:**
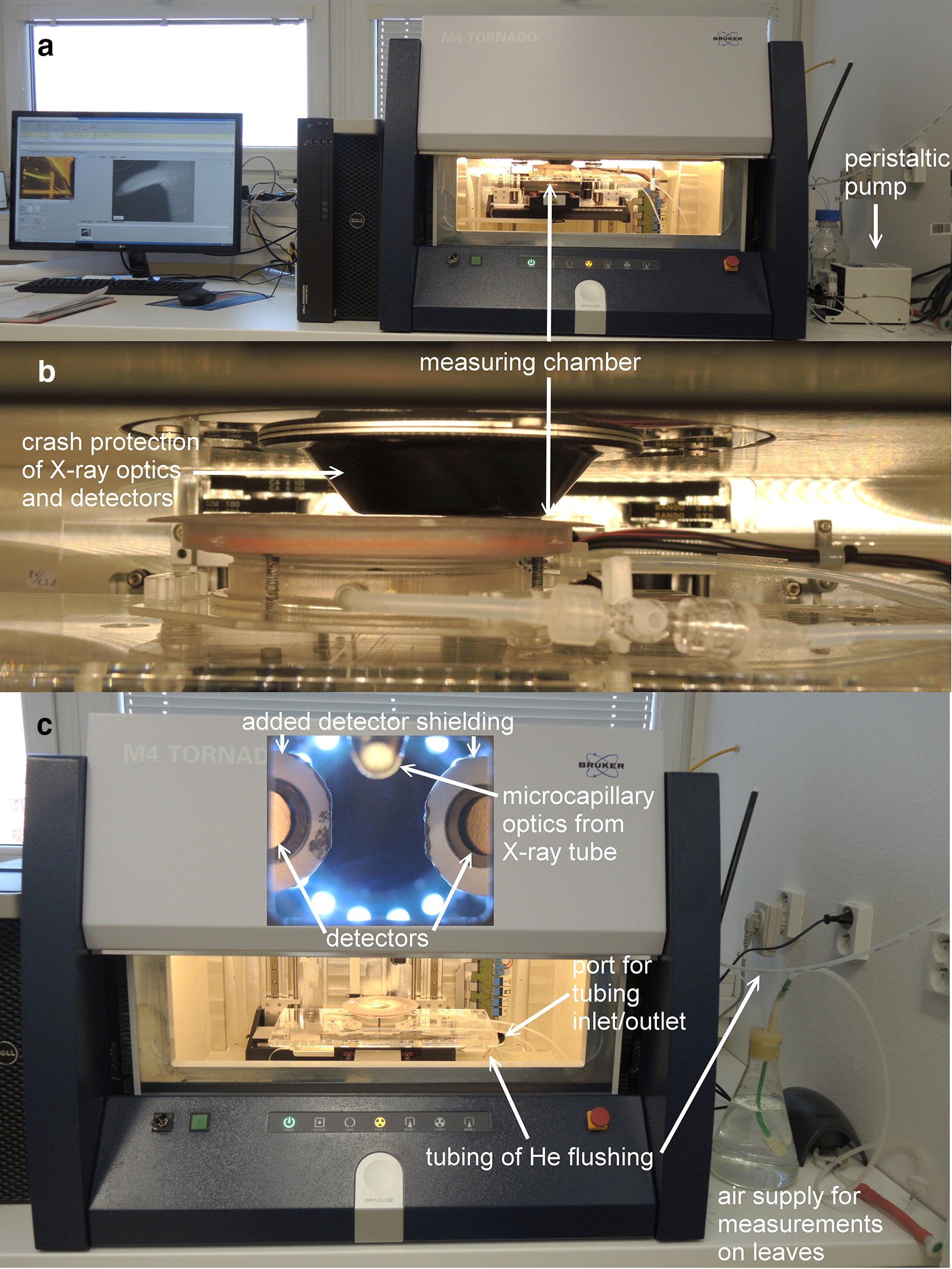
Photos of the µXRF system configuration. **a** Configuration of the whole system, as set up for measurements on roots i.e. with peristaltic pump for liquid media. **b** Close-up view of the in vivo measuring chamber in focus position. **c** Configuration of the system as set up for measurements on leaves i.e. with air supply

Leaves were mounted by putting them onto the measuring chamber, then placing a cotton pad on top, then a plastic disc (with holes for aeration) for gently pressing them. The whole assembly was finally covered with a fine (5 µm pore size) nylon mesh from below, which was stretched by an O-ring (Fig. 2 and Additional file 1: Figure S1). The petiole of the leaf was put into a small water-filled Petri dish in the bottom of the chamber, to keep the leaf hydrated. The chamber was flushed with air. The air was saturated with water by making it bubble in a flask filled with water with an aquaria pump (TetraTec APS 300, Tetra, a Spectrum Brands Company, Melle, Germany) and connected with PTFE tubing to the inside of the µXRF machine enclosure and to the measuring chamber inside (Fig. 3).

Root sections were mounted by putting them in nutrient solution without Zn onto the window of the chamber. Afterwards, a piece of cellophane previously boiled and washed several times in ddH_2_0 to remove any adhering particles was stretched over the roots by the O-ring. The chamber was filled with the same nutrient solution, which was continuously renewed during the measurement via a peristaltic pump (Ismatec REGLO ICC Digital Peristaltic Pump, Cole-Parmer GmbH, Wertheim, Germany) attached to the PTFE tubing outside of the hutch of the µXRF machine (see Fig. 3).

### Chlorophyll fluorescence kinetic measurements

Chlorophyll fluorescence kinetics (for more information see Genty et al. [30]; Baker [31]; Maxwell and Johnson [32]; Stirbet and Govindjee [33]) was measured using a macroscopic fluorescence imaging system with a newly developed ultrafast camera and software for direct imaging of fast fluorescence transients (Photon Systems Instruments (PSI), Brno, Czech Republic). All technical properties of the instrument are described in a recent publication [34]. Measurements of fast chlorophyll fluorescence transient (OJIP) induction kinetics were conducted using a custom-made protocol on dark-adapted leaves as described by Küpper et al. [34]. The frame period was 250 µs, the shutter opening (measuring flash length) was 100 µs and the supersaturating pulses were 4000 µmol m^−2^ s^−1^. The definitions of OJIP parameters as described by [33] were used for calculating maps of OJIP parameters: Φ_Po_—maximum quantum yield of primary PSII photochemistry, Φ_ET2o_—quantum yield of the electron transport flux from Q_A_ to Q_B_, and Φ_RE1o_—quantum yield of the electron transport flux until PSI electron acceptors. Quantum yield refers to stable charge separation or oxygen evolution divided by the number of absorbed photons, or the efficiency of photochemistry (Kalaji et al. [35] and references therein).

Measurements of slow chlorophyll fluorescence induction and quenching kinetics were done according to the protocol described by Küpper et al. [25] but with adaptations for the new measuring device as described by Küpper et al. [34]. In summary, a 1000 ms flash of supersaturating light (4000 µmol m^−2^ s^−1^) for F_m_ was followed by 90 s of darkness, after which F_0_ was measured for 5 s. Then, 100 s of actinic light were applied to analyse the Kautsky induction, and finally 100 s of measurement with no actinic light were used to measure dark relaxation and ${\text{F}}_{0}^{{\prime }}$. During the actinic light exposure (100 µmol m^−2^ s^−1^) and in the dark relaxation period, 600 ms supersaturating flashes were applied for analysis of photochemical (Φ_PSII_-operating efficiency of PSII) and non-photochemical quenching (NPQ = (F_m_ − ${\text{F}}_{\text{m}}^{{\prime }}$)/F_m_). A typical Chl fluorescence trace is shown in Additional file 2: Figure S2.

Chlorophyll fluorescence kinetics of pepper leaves was measured immediately before and immediately after the µXRF measurements, which lasted about 20 h. OJIP (Φ_Po_, Φ_ET2o_ and Φ_RE1o_) and Kautsky parameters (Φ_PSII_ and NPQ) were compared between the X-ray exposed area and adjacent, bordering part of the leaf of the same size as the exposed area. During the µXRF measurements, the camera light was switched off, but the measuring hutch was illuminated.

### X-ray microfluorescence scanning

X-ray fluorescence was measured with a customised M4 TORNADO system (Bruker Nano GmbH, Berlin, Germany). This machine was equipped with two XFlash^®^ silicon drift detectors (type SDD VH50P) able to cope with count rates as high as 310,000 counts per second (cps) with an energy resolution < 145 eV (Mn Kα) according to manufacturer’s parameters (Bruker Nano GmbH, Berlin, Germany). In this work all measurements were done with the Rh tube with a 0.1 mm Be window (Incoatec GmbH, Geesthacht, Germany) and polycapillary optics (IFG Institute for Scientific Instruments, Berlin, Germany). It was operated at 50 kV and 600 µA. The polychromatic incoming flux of the X-ray tube spans over 40 keV with the structure shown in the Additional file 3: Figure S3. All photons with energy above the chosen absorption edge will not be equally absorbed, but with an energy dependent cross section [36]. This means that the beam is not equivalent to a monochromatic beam with the same flux (photons/second). The measuring hutch and the sample stage with the in vivo measuring chamber mounted on it are shown in Fig. 3. A filter composed of 100 µm of Al and 25 µm of Ti was used to flatten the bremsstrahlung in the energy region of the transition metals. A polycapillary lens was used to focus the photons with the highest transmission at 9 keV [37]. Besides the measurement of the transmission of a polycapillary lens shows little variation in the range 5–10 eV (see Fig. 1 in Wolff et al. [38]). The polycapillary lens provided a beam spot size of about 15 µm for the Mn Kα line. The machine was customized for the needs of this project in several ways: (1) At request of the authors the machine was specially fitted with a multilayer mask (Bruker Nano GmbH, Berlin, Germany) on the detectors to reduce spurious counts of Ni, Cu and Zn, resulting in a clean spectrum of Zn and Cu, with a few % contamination of Ni (Additional file 4: Figure S4) [39]. (2) In order to be able to measure samples in vivo, a PTFE tubing was inserted that transports water-saturated air or nutrient solution through the plant measuring chamber. (3) Since the measuring chamber cannot be operated in vacuum, a line for He flushing (inlet and outlet) was installed for enhancing the measurement of low-Z elements. The fluorescence maps were collected for a total time of 3–25 ms per pixel for *N.* *caerulescens*, 150 ms for *A.* *halleri* and 480–720 ms for pepper and soybean.

#### Resolution of the X-ray microfluorescence scanning

When measuring elements from the soft to the hard X-ray range, it is important to remind that the spot size is energy dependent  [37] with a term that is inversely proportional to the energy [24]. Smaller spot sizes are obtained for increasing energies. The size of the spot for transition metals was about 15 µm. To know in how far the spot size is influenced by primary emission filters, the edge of an aluminium foil was scanned without and with an Al–Ti filter (Additional file 5: Figure S5). This test, which also included the absorption and reflectivity effects, showed that the effect on the resolution of the polychromatic X-ray source was negligible.

#### Standards for quantification

The foil standards for calibration were made to include all elements of interest for the current study, with the concentration ratios approximately (order of magnitude) matching the plant samples. Foil standards matching the thickness of the plant leaves were made by dissolving polyvinyl alcohol (PVA, Ultimaker, Geldermalsen, The Netherlands) in double distilled water (20% w/v). PVA was chosen as a matrix because among available polymers it (a) resembles an organic matrix quite well with its formula [CH_2_CH(OH)]_n_, (b) it is very well water soluble and therefore homogenously miscible with the metal solutions until dryness. To this solution, Ca(NO_3_)_2_ and KCl were added from 100 mmol L^−1^ stock solutions, ZnSO_4_ was added from a 1 mol L^−1^ stock solution, Cu-EDTA, FeNa-EDTA, Mn-EDTA and Ni-EDTA were added from 10 mmol L^−1^ stock solutions. The residual volume was filled with water. The resulting final concentrations in the standard stock were: 10% PVA, 0.5 mmol L^−1^ Cu and Mn, 1 mmol L^−1^ Fe, Na and Ni, 5 mmol L^−1^ Ca and K, 5.5 mmol L^−1^ Cl, 11.5 mmol L^−1^ S, 100 mmol L^−1^ Zn (for *N.* *caerulescens*; otherwise 0.5 mmol L^−1^ Zn). The mixture was diluted with 10% PVA to yield standards of different concentrations. The standards were pipetted into a 3D-printed grid of 1 × 1 cm^2^ squares, and left to dry in a cleanroom. Foil layers were stuck to reproduce 200 and 400 μm thickness. The metal content of the foil was determined with ICP-MS. Standards for root samples were polyimide tubings of a diameter close to the root of interest, filled with aqueous solutions of all elements of interest plus 20% glycerol to bring the content of light elements (C, H, O) close to a plant sample.

#### Quantification of a *N. caerulescens* leaf µXRF maps with empirical standards

The fluorescence maps were processed using the software provided with the machine. The fitting method was created with the Xmethod software [40] (Bruker Nano GmbH, Berlin, Germany). The background is determined mathematically by a peak stripping approach that gives a flat background after several cycles. The data was calibrated with the mentioned foil standards to a straight line without offsets. This fully empirical method relies on the assumption that the foil standard resembles the sample matrix as well as the sample thickness. Two different sets of standards were used to match the thickness of about 200 µm for young leaves (at position **0**) and 400 µm for young mature to fully mature leaves (**1**–**4**) of *N.* *caerulescens*, with thicknesses measured with a digital caliper (model Workzone, Dario Markenartikelvertrieb GmbH and Co KG, Hamburg, Germany) and compared to earlier work [18]. The element maps of an entire leaf were collected without sample damage after 4–20 h of measurement with a maximum dwell time of 600 ms in each spot. For full quantification the *N.* *caerulescens* pixels were binned to the lowest amount the software could process (5 × 5 to 9 × 9). The reduced resolution did not affect the conclusions since the epidermal cells in *N.* *caerulescens* have sizes in the range of 100 µm [18, 41]. The µXRF maps of crop plants (*C.* *annum* and *G.* *max*) were not fully quantified, but shown as semi-quantitative maps after spectral deconvolution, in order to avoid the pixel binning and corresponding loss of resolution. Their measured areas were smaller (5 × 5 mm for leaves, and up to 3 × 2 mm for roots) so that the number of pixels was not a problem, but Gaussian smoothing was needed to reduce noise. Gaussian smoothing and assignment of colour scales were done in the Fiji version of ImageJ [42]. Maps were assembled, arranged and labelled using PhotoImpact X3 (Corel Corporation, Ottawa, Canada).

Samples were measured in triplicates for the five developmental stages of *N.* *caerulescens* leaves. Positions **2** and **3** were later averaged due to their similarity. Mean values and standard deviations were calculated in Numbers (macOS High Sierra 10.13.6, Apple, Cupertino, CA, USA). Four leaves from four independently grown plants were measured from *C.* *annuum*. The root of *G.* *max* was measured 2×, once the emerging root hair and once the tip of the lateral root.

### Statistical analysis

Statistics was analysed in Origin Professional (versions 2015 and 2019; Originlab Corporation, Northampton, MA, USA). For analysis of chlorophyll fluorescence parameters that are saturating and do not have normal distribution (Φ_Po,_ Φ_RE1o_, Φ_Et2o_ and Φ_PSII_), the non-parametric Mann–Whitney U test [43] was used for comparison of the X-ray exposed and the adjacent leaf area (same size, bordering the exposed area). One-way repeated measures ANOVA and pair-wise comparison (Bonferroni test) were used to distinguish the effects of X-ray on the same leaf measuring area by comparing the values of NPQ_i1 before and after the X-ray exposure. This test deals with dependent variable which is subjected to repeated measurements—the independence assumption is not considered. The significance level of 0.05 was applied.

## Results

### Characterization of the system and measuring conditions

#### Object with a topography and focusing

Leaf samples show a topography with a structure of main veins, smaller veins, and in the case of *N.* *caerulescens* and *A. halleri* a rippled leaf border. This poses a challenge for focusing. Samples were focused in the middle of the leaf but away from the main vein. To avoid defocusing, the leaves were supported with a 3D-printed Nylon plate with a grid of holes to allow for humidity and air permeation. In a test, the leaf was moved out of focus down, which reduced the detected metal concentration from 2390 mg kg^−1^ in focus to 2262 mg kg^−1^ at 250 µm out of focus and 2225 mg kg^−1^ at 360 µm out of focus. Thus, the change of total metal content in the full image was minimal (at most 7% at 360 µm) since defocusing means that a bigger or smaller solid angle is targeted by the beam.

**Fig. 4:**
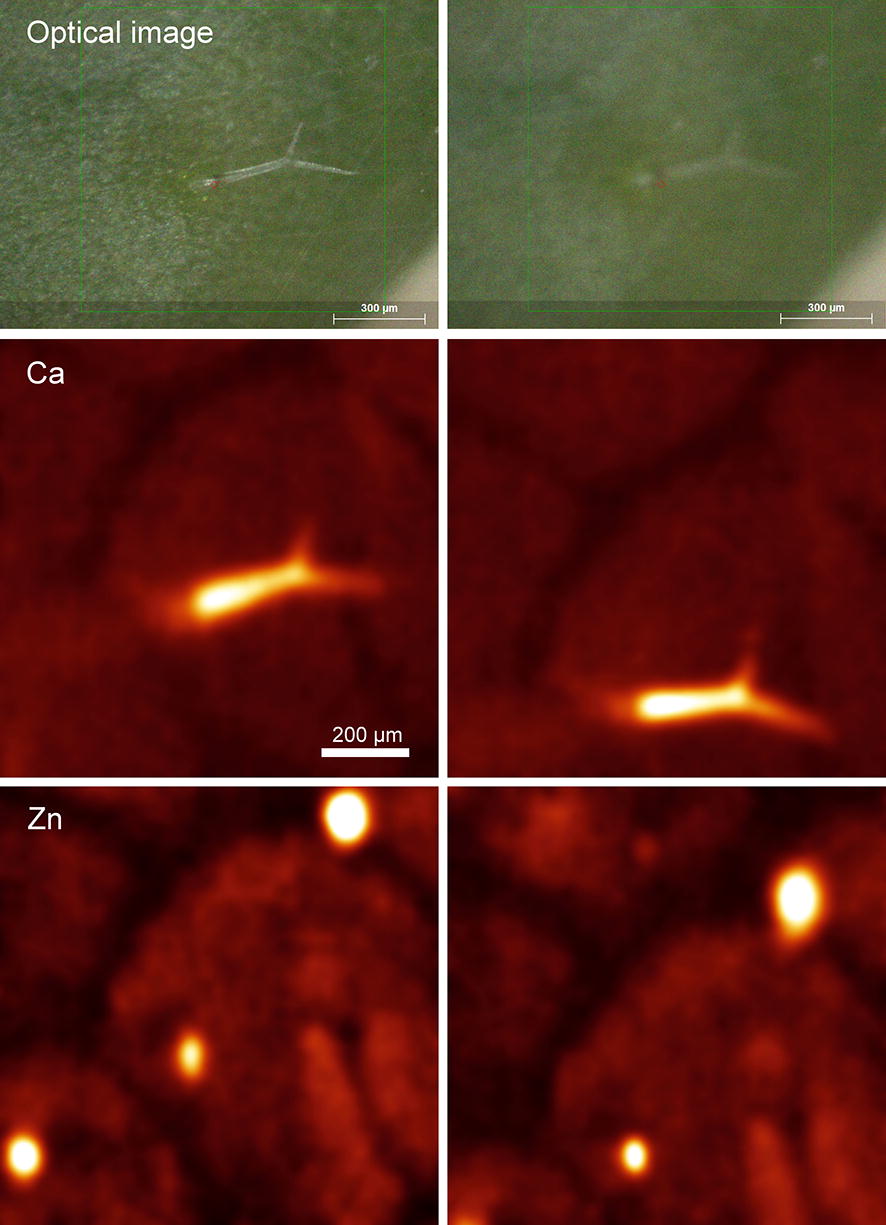
Effect of focusing on the element map of trichomes. A 1 × 1 mm area of an *A. halleri* leaf was measured in a single cycle with 150 ms integration time per point and 4 µm point distance. Measured with He atmosphere in the hutch but air in the measuring chamber. The element maps of this figure are semi-quantitative, obtained by spectral deconvolution but without normalization to a standard. Therefore, the units are detector counts, instead of concentration units

When looking into smaller features like trichomes in *Arabidopsis halleri*, the effects of defocusing became more pronounced (Fig. 4). The loss of focus was much worse for the optical image taken by the camera compared to the loss of focus of features on the element distribution maps, which is due to the different optics. Because of the X-ray excitation coming in an angle (Fig. 3), the defocusing led to a displacement of the µXRF map relative to the optical image. In an object with a topography, the latter effect always leads to some distortion of the µXRF map (Fig. 4). Nevertheless, strong accumulation of Zn was observed in the trichome base, and Ca was observed in the trichome body, while less Ca accumulation was observed in the base and in the trichome tips. This matches earlier work on *A. halleri* leaves by scanning electron microscopy with Energy Dispersive X-Ray Analysis (EDX) element analysis and mapping EDX [44]. Accumulation of calcium phosphate in trichomes of several plant species including *Arabidopsis thaliana* was later analysed in the same way [45]. Similar to our findings, in all cases the lowest Ca accumulation was in the trichome base, allowing elasticity, and the role of trichomes as mechanical sensors was discussed.

#### Measuring leaves in vivo: ensuring the fitness of the specimen

**Fig. 5:**
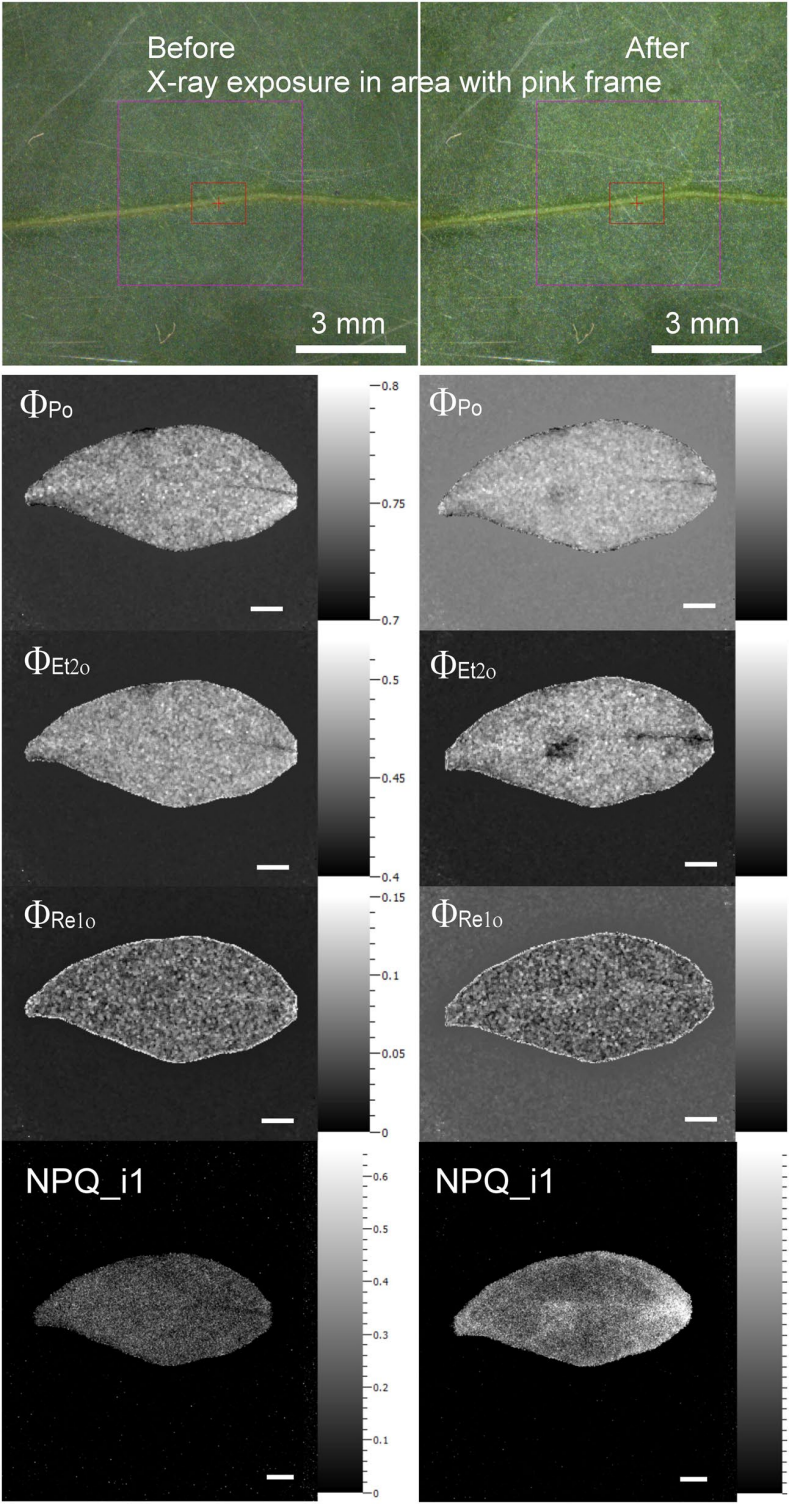
Maps of chlorophyll fluorescence kinetics parameters before and after the µXRF experiment in vivo. A 5 × 5 mm area of a pepper (*C. annuum*, cultivar ‘Kozy Roh’) leaf was measured in a single cycle with 600 ms integration time per point and 15 µm point distance. This resulted in an approx. 20 h total measuring time. The µXRF measurements for these leaves are shown in Fig. 8. Φ_Po_—maximum quantum yield of primary PSII photochemistry, Φ_ET2o_—quantum yield of the electron transport flux from Q_A_ to Q_B_, and Φ_RE1o_—quantum yield of the electron transport flux until PSI electron acceptors, NPQ__i1_ complete non-photochemical quenching (NPQ = (F_m_ − ${\text{F}}_{\text{m}}^{{\prime }}$)/${\text{F}}_{\text{m}}^{{\prime }}$) at the onset of irradiance

No visible signs of damage by the X-rays could be observed on the pepper leaves after about 20 h long measurements (Fig. 5). Fast and slow chlorophyll fluorescence kinetics were measured before and after μXRF measurements and the parameters were calculated for the whole leaves, and on directly X-ray exposed and adjacent area. Overall, on the whole leaf level, no significant differences in photosynthetic parameters could be observed in relation to X-ray exposure (not shown). On the other hand, when the parameters (presented as a ratio before and after the X-ray measurement to exclude differences between the leaves) were compared between the measured area and an adjacent area, a significant decrease in Φ_ET2o_ was observed in the X-ray exposed area, while the effects on Φ_Po_ and Φ_RE1o_ were not so pronounced (Fig. 5, Table 1). Maximal yield of photochemistry (Φ_Po_ = 1 − F_0_/F_p_) was not the most sensitive parameter, as both F_0_ and F_p_ (equals F_m_) slightly decreased as a result of the µXRF measurement.

**Table 1:** Mann–Whitney U test statistics for significant differences at the 0.05 probability level (marked with asterisks sign) in OJIP parameters between X-ray exposed and non-exposed adjacent area (the same size and bordering the exposed area) of pepper leaves (n = 4)

Chl fluorescence parameters	N	Median	U	Z	Asymp. Prob > |U|
Φ_Po_					
Area under X-ray	4	0.985	3	− 1.29904	0.19393
Adjacent area	4	0.993			
Φ_RE1o_					
Area under X-ray	4	1.23	12	1.01036	0.31232
Adjacent area	4	1.1			
Φ_ET2o_					
Area under X-ray	4	0.951	0	− 2.16506	0.03038*
Adjacent area	4	0.991			
Φ_PSII_					
Area under X-ray	4	0.823	4	− 1.01036	0.31232
Adjacent area	4	0.883			

The Z value represents standardized value corrected for the presence of ties in case of similar values which are assigned to the same rank. Asymp. Prob > |U| indicates the asymptotic significance; when it is lower than the statistical threshold, the alternative hypothesis that the two distributions are different, is accepted. Φ_Po_—maximum quantum yield of primary PSII photochemistry, Φ_ET2o_—quantum yield of the electron transport flux from Q_A_ to Q_B_, and Φ_RE1o_—quantum yield of the electron transport flux until PSI electron acceptors according to Stirbet and Govindjee [33]. Φ_PSII-_ operating PSII efficiency according to Küpper et al. [25]

Slow chlorophyll fluorescence kinetics revealed no changes in F_v_/F_m_ in relation to X-ray exposure neither in the whole leaf nor in the X-ray exposed area (not shown). However, the non-photochemical quenching determined here as NPQ = (F_m_ − ${\text{F}}_{\text{m}}^{{\prime }}$)/${\text{F}}_{\text{m}}^{{\prime }}$ during the irradiance phase (NPQ_i1 to NPQ_i3) was increased in the measured leaf area after the X-ray exposure (Fig. 5, significant effect of X-ray exposure determined by one-way repeated ANOVA, Table 2) from 0.089 ± 0.031 to 0.290 ± 0.041 before and after the measurement, respectively. These results indicate the activation of photoprotective mechanisms such as heat dissipation due to decreased electron transport efficiency under X-rays. Operating efficiency of PSII (Φ_PSII_ = (${\text{F}}_{\text{m}}^{{\prime }}$ − ${\text{F}}_{0}^{{\prime }}$)/${\text{F}}_{\text{m}}^{{\prime }}$) was also slightly lower after the X-ray exposure, although not significantly different compared to the adjacent area.

**Table 2:** (A) Results of one-way repeated measures ANOVA and (B) pair-wise comparison for differences in NPQ__i1_ of the same area before and after X-ray exposure (n = 4)

(A) Repeated ANOVA, test of within subjects effect							
		Sum of squares	DF	Mean square	F	Prob > F	
X-ray treatment	Sphericity assumed	0.0804	1	0.0804	11.32322	0.04357*	
Error (xray)	Sphericity assumed	0.0213	3	0.0071			

(B) Pairwise comparison, Bonferroni test							
	Index	Mean difference	Std. error	DF	Prob > |t|	95% LCL	95% UCL
Before/after X-ray treatment	0	− 0.2005	0.05958	3	0.04357*	− 0.39012	− 0.01088

NPQ__i1_—complete non-photochemical quenching ((F_m_ − ${\text{F}}_{\text{m}}^{{\prime }}$)/F_m_) at the onset of irradiance phase (_i1) according to Küpper et al. [25]. Significant differences are emphasized with asterisks

#### Compton scattering images

**Fig. 6:**
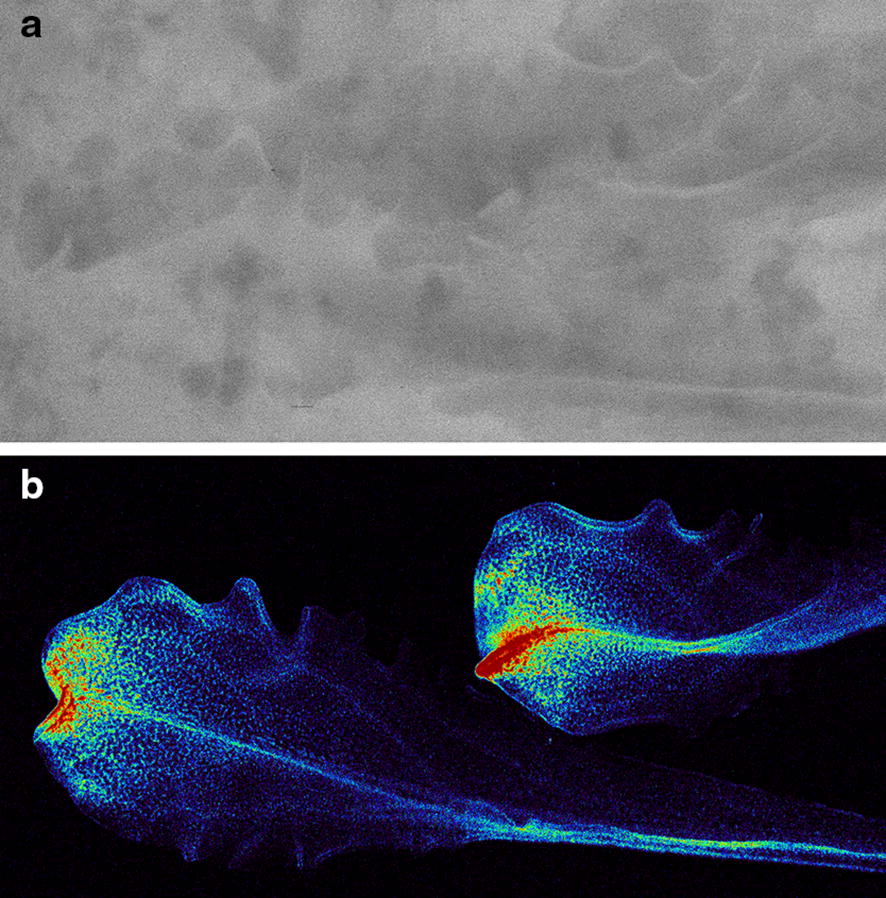
Map of the Compton peak at about 18.9 keV (**a**) corresponding to the Rh Kα line of Rh and Zn fluorescence map (**b**). Both images correspond to the two rosette leaves (position 0) of *N.* *caerulescens*. Point distance is 15 µm and integration time is 10 ms

The map of the Compton line due to the Rh Kα is the most intense and gives information of the inelastic scattering occurring at the supports and in the sample. It is most sensitive to the low Z elements. In the sample mounting used for plants, with water below the leaf for in vivo measurements, the scattering builds up strongly at the materials used as support and hinders the use of the Compton images of the leaves. For low concentration elements, a strong scattering might contaminate the fluorescence map. For those low concentration elements, the comparison of the fluorescence image with the Compton image helps to determine the real nature of the features observed in the fluorescence image (Fig. 6).

#### Quantification with M4 and Xmethod on a certified liquid standard

A test of the quantification was done on a certified liquid standard for ICP-MS (standard solution VI, Merck KGaA Darmstadt Germany) using as calibration three dilutions of the liquid reference material of the synchrotron tomography [46]. Therefore, the certified liquid standard allowed a “quality test” of the quantification with the benchtop X-ray machine. The results are displayed in Tables 3 and 4.

**Table 3:** Concentrations of Mn, Fe, Ni, Cu and Zn for the certified liquid standard

Sample	Mn [mg kg^−1^]	Fe [mg kg^−1^]	Ni [mg kg^−1^]	Cu [mg kg^−1^]	Zn [mg kg^−1^]
µXRF result	6.85	90.60	8.48	8.47	87.90
Certificate of analysis (manufacturer)	9.9 ± 0.5	100 ± 5	9.9 ± 0.5	9.9 ± 0.5	99 ± 5
RSE %	18.21	4.93	7.73	7.78	5.94

Data from: (a) µXRF quantification, (b) datasheet of manufacturer (Merck). Data were collected for an area and the average of the pixel grey values was used. The relative standard error percentage (SE/mean %, with n = 2 and SE = STDEV/sqrt(n)) shows the difference between the µXRF result and the certificate of analysis

**Table 4:** Calibration for the liquid standards: content in standard sample measured with ICP-MS and the calculated value from the µXRF calibration

Standard samples	K [mg kg^−1^]	Ca [mg kg^−1^]	Mn [mg kg^−1^]	Fe [mg kg^−1^]	Ni [mg kg^−1^]	Cu [mg kg^−1^]	Zn [mg kg^−1^]
Std 1							
ICP-MS	195	200	275	279	293	318	327
µXRF	194	203	274	272	294	315	327
Std 2							
ICP-MS	98	100	137	140	147	159	164
µXRF	101	86	140	154	146	166	162
Std 3							
ICP-MS	49	50	69	70	73	79	82
µXRF	47	61	67	62	74	75	82
Std 1							
RSE %	0.26	0.74	0.18	1.27	0.17	0.47	0.26
Std 2							
RSE %	1.51	7.53	1.08	4.76	0.34	2.15	1.51
Std 3							
RSE %	2.08	9.91	1.47	6.06	0.68	2.60	0.00

The relative standard error percentage (SE/mean  %, with n = 2 and SE = STDEV/sqrt(n)) shows the difference between the µXRF result and the ICP-MS

#### Quantification with M4 and Xmethod using foil standards

The foil standards reproduce at best the leaf matrix and its thickness. Therefore, all final quantifications of leaf measurements were done with the foil standards. Their concentrations measured by ICP–sfMS are shown in Table 5. The calibration parameters of the µXRF measurement are shown in Tables 6 and 7.

**Table 5:** Quantification of the foil standards with ICP-MS: PVA 1-XX indicates the dilution in XX parts

	Mn [mg kg^−1^]	Fe [mg kg^−1^]	Ni [mg kg^−1^]	Cu [mg kg^−1^]	Zn [mg kg^−1^]
Reagent Blank1	0	0	0	0	0
Reagent Blank2	0	0	0	0	0
PVA zero	1	2	0	1	1
PVA 1-100	3	4	16	4	529
PVA 1-50	3	5	19	5	634
PVA 1-30	5	9	34	8	1108
PVA 1-20	8	13	56	13	1835
PVA 1-10	15	22	104	24	3352
PVA 1-5	39	56	264	59	8719
PVA 1-3	63	91	434	94	14,482
PVA 1-2	92	131	630	137	27,528
PVA 1-1	167	241	1158	244	52,207

**Table 6:** Concentrations in the foil **1-1,** thickness 450 µm, determined by: (a) µXRF, (b) ICP-MS

Foil **1-1**	K [mg kg^−1^]	Ca [mg kg^−1^]	Mn [mg kg^−1^]	Fe [mg kg^−1^]	Ni [mg kg^−1^]	Cu [mg kg^−1^]	Zn [mg kg^−1^]
µXRF	1119	1444	182	265	1161	276	52,763
ICP-MS	1091	1378	167	241	1158	244	52,207
RSE %	1.27	2.34	4.30	4.74	0.13	6.15	0.53

The relative standard error percentage (SE/mean %, with n = 2 and SE = STDEV/sqrt(n)) shows the difference between the µXRF result and the ICP-MS

**Table 7:** Calibration for the foil standards: content in standard sample measured with ICP-MS and its calibrated value from µXRF

Standard samples	K [mg kg^−1^]	Ca [mg kg^−1^]	Mn [mg kg^−1^]	Fe [mg kg^−1^]	Ni [mg kg^−1^]	Cu [mg kg^−1^]	Zn [mg kg^−1^]
Std 1							
ICP-MS	1091	1378	167	241	1158	244	52,207
µXRF	1119	1444	181	262	1158	277	52,978
Std 2							
ICP-MS	615	772	92	131	630	137	27,528
µXRF	513	628	89	120	647	125	25,418
Std 3							
ICP-MS	430	544	63	91	434	94	14,482
µXRF	441	530	52	78	402	73	15,120
Std 4							
ICP-MS	51	68	5	9	34	8	1108
µXRF	72	129	0	12	49	0	1858
Std 5							
ICP-MS	36	74	3	4	16	4	529
µXRF	78	105	0	0	16	0	480
Std 1							
RSE %	1.27	2.34	4.02	4.17	0.00	6.33	0.73
Std 2							
RSE %	9.04	10.29	1.66	4.38	1.33	4.58	3.99
Std 3							
RSE %	1.26	1.30	9.57	7.69	3.83	12.57	2.16
Std 4							
RSE %	17.07	30.96	100.00	14.29	18.07	100.00	25.29
Std 5							
RSE %	36.84	17.32	100.00	100.00	0.00	100.00	4.86

The relative standard error percentage (SE/mean  %, with n = 2 and SE = STDEV/sqrt(n)) shows the difference between the µXRF result and the ICP-MS

#### Limits of detection

The limits of detection (LOD) for both the liquid and foil standards were calculated with the formula [47]:

$$ LOD = \frac{{3*c*\sqrt {2*N_{bkg} } }}{{N_{net} }} $$

with c: concentration, N_bkg_: background counts, and N_net_: peak counts. The results are shown in Table 8.

**Table 8:** Limits of detection (LOD) for the liquid reference from Merk and for the reference foil (foil with 450 µm thickness)

	K	Ca	Mn	Fe	Ni	Cu	Zn
Standard from Merck	–	–	17	15	8	13	13
Foil with 450 µm thickness	494	269	24	16	19	104	86

### Application examples

#### Flat object 1: metal distribution and leaf development stage in *N.* *caerulescens*

**Fig. 7:**
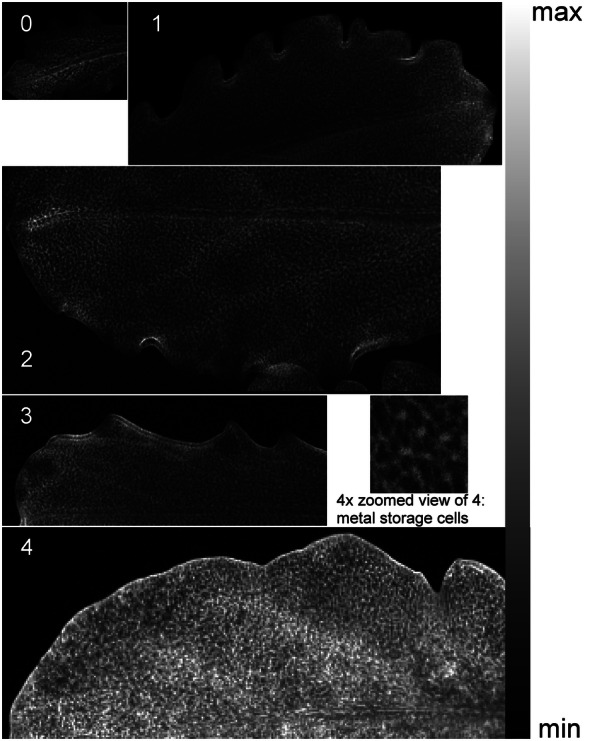
Quantified fluorescence maps of the Zn K-edge of *N.* *caerulescens* for leaves at different stages of development from rosette (0) to the mature leaves at the base of the plant (4). The grey scale on the right side represents the concentrations from 0 (black) to maximum (white). White corresponds to: 12,605 mg kg^−1^ for the leaf at position 4 (base), 3389 mg kg^−1^ for position 3, 3041 mg kg^−1^ for pos 2, 2602 mg kg^−1^ for pos 1, and 2724 mg kg^−1^ for the leaf at position 0 (rosette). These values are corrected to match the difference in mass between the fresh leaves in µXRF with dry mass in ICPMS. Point distance is 15 µm and integration times are: 25 ms for 0, 3 ms for (1)–(4). Pixels are averaged (5 × 5 to 9 × 9) in the quantified images due to limitations of the software to deal with high pixel number. The full resolution zoom shows the epidermal metal storage cells [18]

Figure 7 shows the fluorescence images for Zn distribution for five different development stages of the *N.* *caerulescens* leaves, from the rosette apical meristem—young leaf (position **0**) to a mature, but not senescent, leaf at the base of the plant (position **4**). The high-resolution image shows a structure of Zn distribution that resembles the tissue structure and Ni distribution seen for the related *N.* *goesingense* (= *Thlaspi goesingense*) in Küpper et al. [48], as well Zn distribution in *N. caerulescens* [18]. In both species the epidermal cells have a wide range of sizes, with large cells (“metal storage cells”) accumulating the highest Zn and Ni content far away from stomata, and the smaller cells around the stomatal guard cells.

Young, actively growing leaves of *N.* *caerulescens* already accumulated high amounts of Zn. Leaves taken at the rosette apical meristem accumulated about 3000 mg kg^−1^ Zn. The Zn concentration increased in a non-linear manner with leaf age, reaching about 7500 mg kg^−1^ in the oldest mature leaves. Leaf size and metal content variability were important at all stages of the plant development. Quantification of Zn fluorescence was compared with ICP-MS analysis for individual leaves (Table 9). On average, the mean values for Zn content between XRF and ICP-MS measurements differed up to 20%, except for older leaves where the difference was slightly higher. Regarding Zn distribution, in young leaves the highest accumulation was observed towards the tip and in the veins. The oldest leaf had a more homogeneous Zn distribution, and the highest concentration in the veins and rims. As both, the exciting X-ray beam and the Zn Kα fluorescence, penetrates through the leaf, in such 2D-scans of intact leaves it is not possible to distinguish from which depth inside the leaf the signal originates. It could be epidermis or mesophyll for regions between veins, and epidermis above veins or epidermis itself when looking at a vein. Earlier work using SEM–EDX on frozen-hydrated leaf sections showed that Zn is mostly in the epidermis [18], but in that work the macroscopic distribution (tip vs. base, veins vs. interveinal areas) could not be analysed. A study of metal distribution of *N. caerulescens* using laser ablation-inductively coupled plasma mass spectrometry (LA-ICP-MS) showed gradual increase in Zn accumulation from the base to the leaf tip, but for the oldest leaf [49]. Comparison with the current work is difficult since the growing conditions were different (1/5 of Hoagland’s solution, and fivefold more Zn than in this work) and the leaf was scanned by ablation for 15 h without cryoprotection.

**Table 9:** Zn concentrantions in the leaves in positions **0**–**4** from top to bottom with **0**: rosette and **4**: mature leaf, sampled from three plants

Sample	Pos **0** µXRF [mg kg^−1^]	Pos **0** ICPMS [mg kg^−1^]	Pos **1** µXRF [mg kg^−1^]	Pos **1** ICPMS [mg kg^−1^]	Pos **2** and **3** µXRF [mg kg^−1^]	Pos **2** and **3** ICPMS [mg kg^−1^]	Pos **4** µXRF [mg kg^−1^]	Pos **4** ICPMS [mg kg^−1^]
Mean	2840	3181	2271	2763	3496	3453	7838	4250
Standard error	345	615	245	980	308	503	1438	872
RSE %	5.66		9.77		0.62		29.68	

Please note that the same leaves were used for µXRF and ICP-MS, but entire leaves were digested for ICP-MS, not only the area on them that was measured by µXRF. These values are corrected to match the difference in mass between the fresh leaves in µXRF with dry mass in ICPMS. The relative standard error percentage (SE/mean  %, with n = 2 and SE = STDEV/sqrt(n)) shows the difference between the µXRF result and the ICP-MS

#### Flat object 2: element distribution in the leaves of pepper

**Fig. 8:**
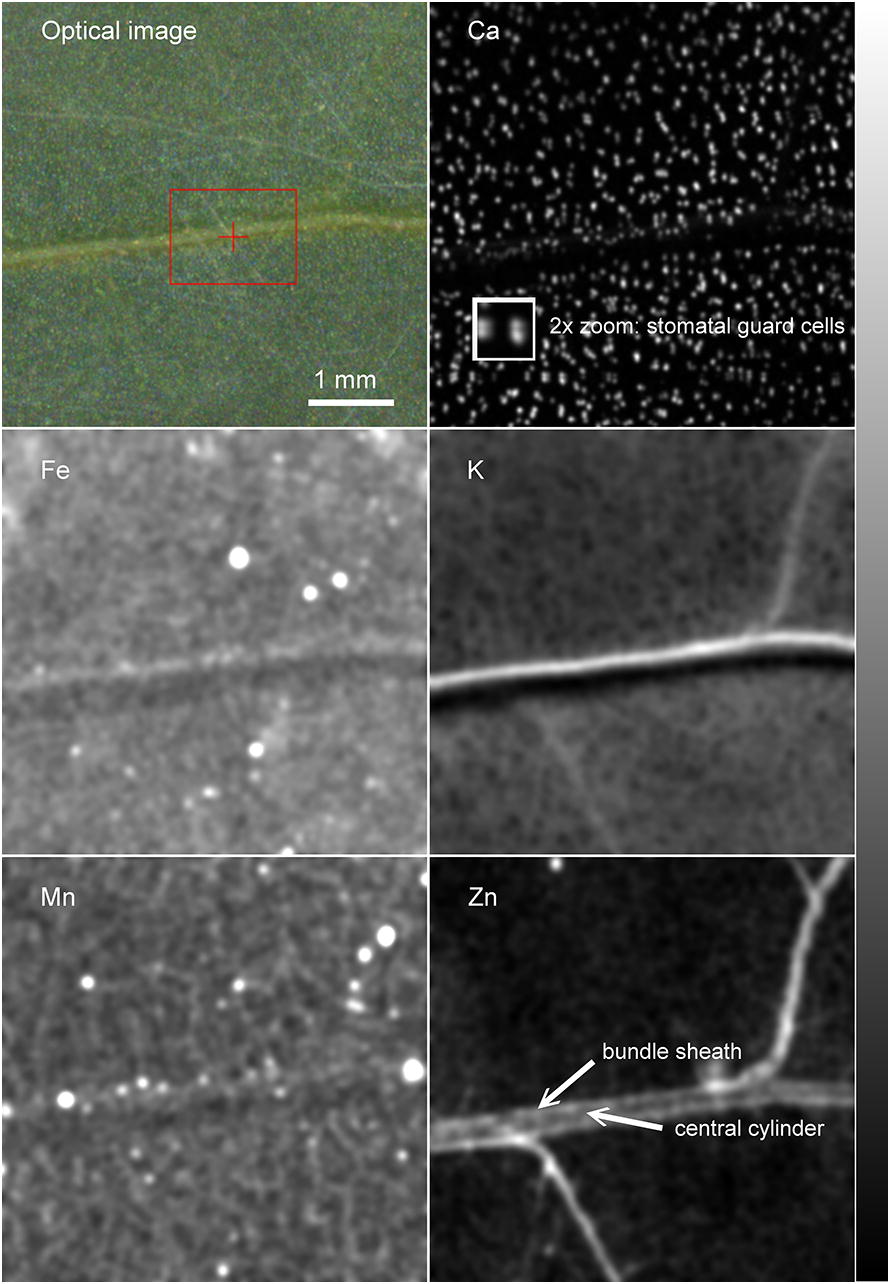
Metal distribution in a pepper leaf (*C.* *annuum*, cultivar ‘Kozy Roh’). The leaf was measured in a single cycle with 600 ms integration time per point and 15 µm point distance. Measured with He atmosphere in the hutch but air in the measuring chamber. Maps of photosynthetic parameters for the same leaf before and after radiation are shown in Fig. 5. Gaussian smoothing was applied to all element maps (sigma = 1 for Ca, sigma = 3 for all other elements). The element maps of this figure are semi-quantitative (0 = black, maximum = white), obtained by spectral deconvolution but without normalization to a standard. Images are then in detector counts instead of concentration units. The zoomed image of the Ca map shows the epidermal guard cells, which are known to contain high Ca, confirming the high resolution of the µXRF measurement

High sensitivity for trace elements was achieved after comprehensive system optimization as described in the Methods section. As a result, micronutrients could be analysed in natural abundance in crop plants, as shown by the analysis of a pepper leaf (Fig. 8). The multielement analysis by ICP-MS of leaves from the same plants is shown in Additional file 6: Table S1. The signals of Fe, Mn and Zn were high enough to yield high-resolution maps, so that their distribution could be compared to that of the more abundant macronutrients, Ca and K (Fig. 8). While Ca was homogeneously distributed in the mesophyll and localized mostly in stomatal guard cells of the epidermis (see zoomed image), with no enrichment in the veins, Zn was predominately accumulated in the veins. In the larger veins most of the Zn accumulated in the bundle sheath cells (see labels in Fig. 8). Only very low Zn concentrations were found in the mesophyll. Manganese distribution was opposite to that of Zn: no accumulation in the midrib, but higher accumulation in the smaller veins and the mesophyll, with a distinct pattern that might reflect the structure of the spongy mesophyll. Fe and K were in between these two extremes, with somewhat higher accumulation in the veins than in the mesophyll.

#### Cylindrical object: soybean roots

**Fig. 9:**
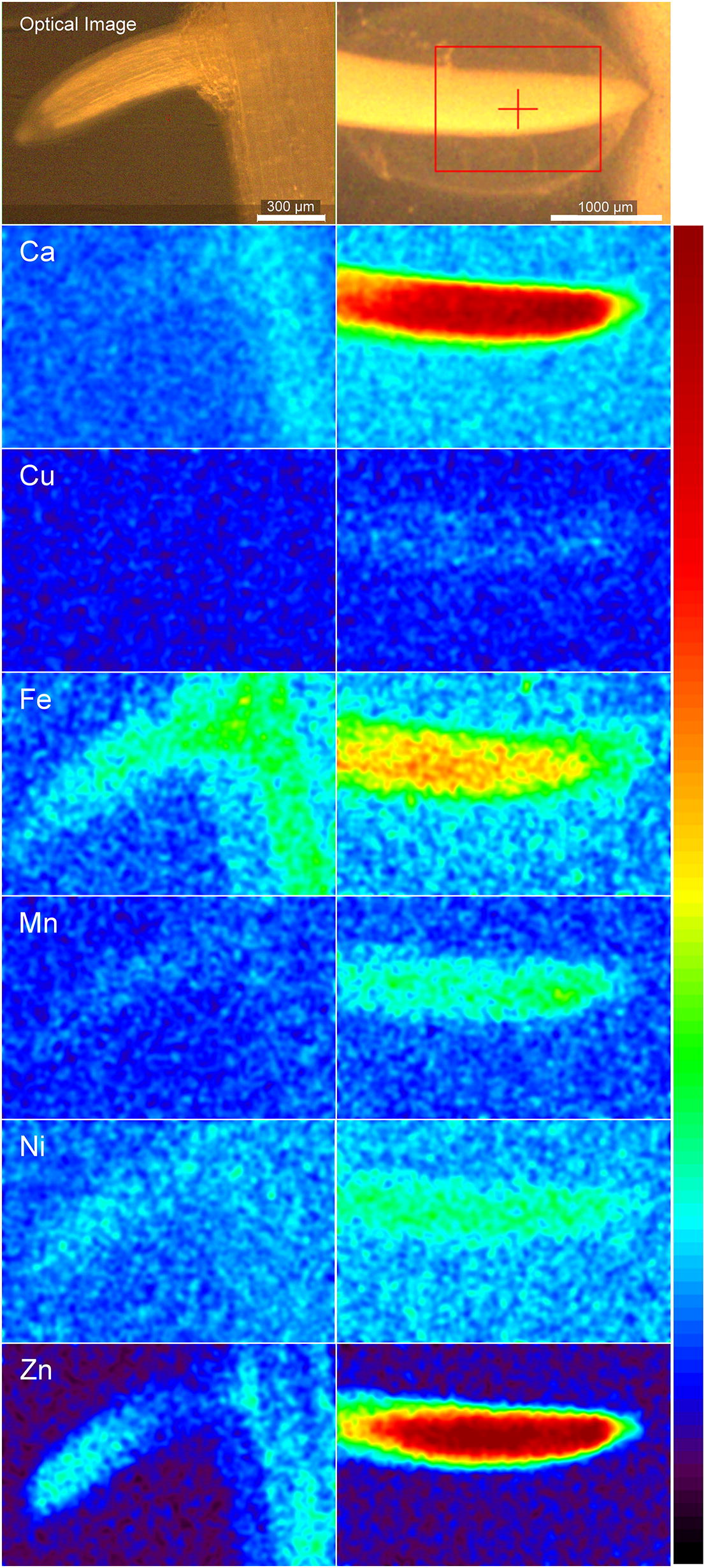
Metal distribution in a root of soybean (*G.* *max*, cultivar ‘Galina’). **a** Tip of the lateral roots. The root was measured 120 times (cycles) by summing up the counts for each pixel, each cycle with 6 ms integration time per point and point distance of 8 µm. The machine enclosure had a He atmosphere but measuring chamber was with air to keep the root alive. Gaussian smoothing was applied to all element maps (sigma = 3 for all elements). **b** Older part of the same lateral root with an emerging root hair. This was measured with 80 cycles, each with 6 ms integration time per point and 4 µm point distance. Gaussian smoothing was applied to all element maps (sigma = 3 for all elements). The element maps of this figure are semi-quantitative, obtained by spectral deconvolution but without normalization to a standard. Images are in detector counts instead of concentration units

In fresh, living state, roots turned out to be the most challenging plant organs for analysis with µXRF, not only for the benchtop system presented here (Fig. 9) but also in recent synchrotron beamtimes of the team (not shown). There are two main reasons why roots of non-accumulator plants at non-toxic trace metal concentrations are difficult samples for this technique. First, the water content is much higher than that of leaves or seeds. Thus, their ratio of fresh mass to dry mass is very high (about 20 for soybean roots), and the high metal concentrations usually reported on a dry mass basis translate to very small concentrations in fresh living tissue. The concentrations measured by ICP-MS on dried homogenised roots of soybean are shown in Additional file 6: Table S1. This could be overcome by the sensitivity optimization of the current system, as shown in Fig. 9. In the lateral root tip, all known micronutrients (Cu, Fe, Mn, Ni, Zn) could be visualized with reasonable signal/noise ratio (Fig. 9a). The concentration of Cu in the emerging root hair became too low to be visualized, but the other micronutrients remained well measurable (Fig. 9b). In the root tip the signal intensity for all elements was the lowest in the root cap; Zn was mostly accumulated in the meristem and in the endodermis of the main root, while Fe accumulation was more pronounced in the elongation zone. Secondly, roots are cylindrical, which causes problems for quantification in a benchtop µXRF system, because only projections from 2D scanning are possible, while tomography is not feasible with the current equipment.

## Discussion

This work showed that the customized version of M4 TORNADO is suited for measuring samples in vivo, contrary to standard benchtop µXRF machines. This was made possible by four key factors:

1. The choice of the maximal effective detector area commercially offered, i.e. 2 × 60 mm^2^ active area. This maximizes the solid angle and thus the efficiency of fluorescence detection, minimizing the X-ray irradiation that is needed for obtaining a good signal to noise ratio. Primary emission filters may be employed to absorb unwanted regions of the polychromatic exciting X-ray spectrum. The large area of the detectors facilitates the collection of spurious counts from scattering in other parts of the machine. This unwanted effect was minimized by the additional shielding in the detector.
2. The construction and implementation of a chamber for measuring plant samples under physiological conditions such as flow of water-saturated air or liquid media. The sample chamber is tightly enclosed to avoid moisture on beryllium detector windows or in the electronics. Thus, since the sample is enclosed, the hutch of the µXRF may even be kept in He atmosphere. It is also possible to measure in air, that is, in the living conditions for the plant. The energy transfer to the sample by the photon flux is lower than in the case of electrons, minimizing the heating. The special mounting in a special measuring cell with liquid and air flow also serves to cool the sample. The compatibility of this chamber with microscopic and macroscopic imaging of UV/VIS fluorescence allows for direct correlation of element distribution with measurements of physiological parameters on the same sample. In the current study, this was used to assess the effect of the X-ray exposure on photosynthesis via fast imaging measurements of Chl fluorescence induction. In principle, measuring detached leaves (or roots) is certainly not as ideal “in vivo” as measuring on an intact plant. But for most plant species the measurement of an intact entire plant in any XRF or UV/VIS/NIR fluorescence imaging device is not possible simply due to size restrictions, so that keeping a detached organ as vital as possible as in the current work is the closest possible match to the ideal “in vivo” condition.
3. Optimization of the machine and the measuring chamber used for analysis of leaves of a non-accumulator plant, pepper, for about 20 h in the µXRF machine ensured that the leaf remained intact without severe damage of photosynthetic apparatus. This was confirmed by comparing photosynthetic parameters before and after the X-ray exposure. In addition, we observed higher sensitivity of PSII to radiation than PSI. To our knowledge, the studies of the effects of X-rays on plant performance are scarce, but could become important for the growth of plants on space stations. In experiments aimed at revealing X-ray CT of living rose plants, performed in a synchrotron [50], significant structural damage was observed. In a later similar study on more species, strong RNA and membrane damage was found [51]. Dose-dependent X-ray inhibition of photosynthetic oxygen evolution was observed in *Chlorella pyrenoidosa* [52]. In bean plants, in an experiment aimed at determining stress to plants in Bioregenerative Life Support Systems (BLSS) of space stations, only a small reduction of 1,5-bisphosphate carboxylase (Rubisco) activity was found [53]. In summary, comparing out results with past studies, it is clear that assays of beam damage as done by Chl fluorescence kinetics in the current work are crucial for verifying that X-ray exposure of plants does not damage these study subjects too much.
4. In terms of metal localization, we demonstrate that a benchtop system is able to yield high-resolution maps of micronutrients in intact leaves and roots of non-accumulator crop plants (pepper and soybean). The achieved spatial resolution is enough to provide information of metal distribution on the tissue level, and in some cases even individual cells can be seen. In leaves, for example, it can show the metal distribution inside trichomes, the Ca accumulation in stomatal guard cells, the Zn accumulation in epidermal metal storage cells and the bundle sheath of veins. This vastly extends the application of the system in plant research, because not all processes of metal uptake, translocation and sequestration studied in hyperaccumulator plants can be directly related to non-hyperaccumulators. In earlier systems, researchers tried to overcome the sensitivity limitation by immersing roots in extremely high trace metal concentrations, 900 µmol L^−1^ each for Fe and Mn [22]. Such concentrations never occur in vivo and are well-known to be highly toxic, so that the value of such measurements was highly questionable. With the current system, in contrast, plant nutrition and sublethal toxicity can be measured under fully realistic growth conditions. This is decisive for the use of such systems in plant breeding, assessment of the quality and safety of food, as well as environmental studies. Subcellular resolution can only be attained at a nanoprobe beamline in a 3rd generation synchrotron [54]. This cannot be done in vivo, due to the strong radiation damage, and it requires shock frozen samples [55]. Therefore, only a benchtop system as shown here near to suitable cultivation facilities allows for direct comparison of element distribution in tissues with other physiological parameters, e.g. photosynthesis. Further, in many cases it is impossible to know when preparing a synchrotron sample which part of a larger plant organ like a leaf is actually representative for the whole organ, or which part is the most interesting in terms of metal distribution. Here, a benchtop system can provide the information that is necessary for an informed decision.

Due to the lower metal content in roots than in leaves, imaging of metal distribution in roots has been done using synchrotron radiation (see Table 6 in review of de Carvalho et al. [56], and review of Kopittke et al. [57]) but not with benchtop machines. For example, using 2D scans with synchrotron light with a beam size of 10 × 7 µm, Sarret et al. [58] studied the metal distribution in mycorrhized roots of tomato grown in heavily polluted soil. Zn and Cu were highly concentrated in the whole root while Fe and Mn were more concentrated at the surface of the root. All four elements were in high concentration at the fungal filaments. Using synchrotron microtomography with resolution 15–20 µm, Terzano et al. [59] found that Zn in roots of rocket plants grown on compost amended soil is mainly in the endodermis and xylem vessels, while plants grown in untreated soils showed a more homogeneous Zn distribution. However, soils were artificially polluted with very high amounts of Zn (0.1 mol L^−1^). The level of resolution achieved in these synchrotron works on crop plants, scanning areas in the millimetre range, compares well with the results of this work with a benchtop machine for hydroponics plants treated with 100 µmol L^−1^ Zn.

The polycapillary optics has the best transmission properties in the region of the K-edges of the transition metals at about 9 keV [37]. The bremsstrahlung radiation from the Rh Kα gets absorbed in the sample matrix for thick leaves, with different layers of cells getting a different excitation profile. However, X-rays have a longer penetration depth than electrons. With water as the main element in the matrix of biological samples, and an excitation spectrum mainly due to Rh Kα, and the bremsstrahlung tail that is filtered in the transition metals K-edge region, it is expected that the epidermis cells contribute the most to the image. For heavier elements like Zn, also the deeper layers of the leaf are completely penetrated, while for light elements like Ca the X-ray fluorescence from deeper layers becomes highly attenuated.

For the spatial resolution both the step size and counting time have an influence. Manufacturer’s test with the Siemens star Cr map shows that 5 µm step with 50 ms accurately reproduces the star in high resolution [60]. Plant samples have much lower concentrations, for which we used from 720 ms integration time per pixel for the lowest concentrations in non-accumulators to 3 ms for the highest concentration samples in hyperaccumulators. Oversampling further improved the visualization of low concentration elements as the density of scanning lines was increased 4×, i.e. the same area was scanned 4x longer. In the current study this strategy was successfully applied as single cycle scanning for the *A.* *halleri* trichomes and as multi-cycle scanning for the soybean roots. The advantage of measuring a single low speed cycle compared to measuring many cycles with higher stage speed is the complete removal of artefacts that occur when a living sample slightly changes shape during the measurement e.g. due to growth processes or movement.

When comparing the collection times with those of electron microscopy (like EDX) it should be kept in mind that the X-ray fluorescence at the energies of the K- edges of transition metals and lighter elements is less efficient than Auger electrons due to the lower yield for fluorescence [61]. Detection limits of XRF, however, are better than for EDX because no bremsstrahlung background is generated in the sample, and the bremsstrahlung generated in the X-ray tube can be removed by primary emission filters, leading to the trace element sensitivity shown in the current work. Further, the problem of long collection times of XRF can be amended with large-area detectors, as done in the current work.

Plant leaves and roots are objects with inhomogeneous surface. Flattening of very curvy leaves like in *N.* *caerulescens* is not always possible without damage, and trichomes like on *A.* *halleri* leaves cannot be flattened anyhow. Mounting over a layer of perforated print foil or perforated polycarbonate thin layer, improved the flatness of the specimen. However, moving the specimen out of focus up to the hundredths of microns gave a variation in concentration of about 10% for homogeneous areas. The most significant loss might be of resolution of features on the specimen like trichomes, which was tested with *A.* *halleri* leaves, and showed surprisingly low defocussing of the µXRF maps. The excitation of the sample from an angle, however, led to an optical shift of out-of-focus parts of the sample, in effect leading to a distortion of the map. This has to be kept in mind when comparing element localizations on non-flat samples.

We observed high variability in Zn content between the leaves of the same development stage of *N.* *caerulescens* (Table 9). In this context it is important to mention that the empirical standard should match the thickness of the leaf to yield a correct quantification. A lower count rate at a thinner area may be accounted as having a lower concentration. Therefore, different standards are needed and were used for mature vs. young leaves.

Both the certified liquid standard (Table 3), and the laboratory-made liquid and foil standards mimicking the carbon composition of the leaf samples (Tables 4, 6 and 7), validated the elemental concentration determination. The relative standard error percentage (RSE  %) in the certified standard (Table 3) showed that for elements in a concentration above the LOD (several tenths mg kg^−1^, see Table 8) the RSE % was below 10%. The laboratory-made standards furthemore provided a reasonable approximation to the metal distribution content for elements within the LODs (Tables 6 and 8 for the LODs). For Ca and K the higher RSE % may be linked to the background-related difficulties in measuring them in ICP-MS.

The LOD shows higher values for the lab made foil standards. In the case of K and Ca, the sensitivity could be increased by increasing the net counts when measuring in He atmosphere instead of air.

The highest Zn content was detected in the oldest mature leaves (development stage **4**) at the base of the rosette (about 3 times higher that of the youngest leaves). In a previous extensive study [20] the amount of Zn in young and mature leaves of *N.* *caerulescens* was similar in young plants under 100 μmol L^−1^ Zn, and the same was observed here for development stages **0**–**3**. It should be considered that ICP-MS gives the total metal concentration in the leaf after digestion. Apart from that, the total metal concentration can be different from the concentrations determined by the microfluorescence considering distribution variability within a leaf. This uneven distribution is particularly visible in hyperaccumulator plants, where even a high Zn concentration at the rim of the leaves can be found (Fig. 7). Older leaves show a more homogeneous Zn distribution than younger leaves, maybe as a result of reaching a threshold for accumulation in the tip, with all subsequent metal being accumulated in the rest of the blade.

## Conclusions

The work shows that an optimized benchtop machine can be used for analyses of the metal distribution in plants in vivo. Together with appropriate quantification methods, it allows determination of metal distribution and quantification in a reliable manner in intact, living plant leaves and roots with a sensitivity of a tenths of mg kg^−1^ and a space resolution down to about 15 µm. The spatial resolution achievable is enough to provide information of metal distribution on the tissue level, and in some cases even in individual cells. In the leaves we showed Ca accumulation in stomatal guard cells, Zn accumulation in epidermal metal storage cells and the bundle sheath of veins, and Ca and Zn distribution in trichomes. Truly non-destructive in vivo measurement of micronutrients became possible not only by the optimization of the µXRF machine itself, but also by the construction of a measuring chamber in which plant organs like leaves or roots can be kept in physiological conditions throughout long measurements. With this chamber, it became furthermore possible to correlate element distribution of the specimen with maps of parameters of photosynthesis via fluorescence kinetic measurements, which demonstrated high sample vitality even after a long X-ray irradiation (up to 20 h measurements for the entire specimen, up to 600 ms per spot) needed to obtain the micronutrient distribution in non-accumulator plant. The long counting time disadvantage is surpassed by the possibility to measure the specimen at any growth stage and being able to measure a number of replicates, both impossible to achieve in a synchrotron where beamtime is limited. In summary, the optimised benchtop machine opens the possibility to measure in the laboratory micronutrient distribution along the plant growth cycle and in response to abiotic and biotic stress.

## Supplementary information

**Additional file 1: Figure S1.** Scheme of the sample mounting in the measuring chamber for both the µXRF and chlorophyll fluorescence kinetics measurements. The specimen is gently pressed against a printer foil window with a cotton pad (to avoid damage) and a 3-D printed polycarbonate plate to keep the specimen straight. A nylon mesh is used to press everything against the window by fixing it with an O-ring around the cover lid rim. The holes grid in the polycarbonate plate and the permeability of fine nylon mesh ensures the supply of air and humidity to the specimen.

**Additional file 2: Figure S2.** Chlorophyll fluorescence kinetic measurements of a pepper leaf. The intensity of the supersaturating flashes was 3500 μmol m^−2^ s^−1^ and actinic light was 100 μmol m^−2^ s^−1^. Intensities of F_0_ (minimal Chl fluorescence of the dark-adapted leaf). Fm (maximal Chl fluorescence of the dark-adapted leaf), F_m__i1′ (maximal Chl fluorescence under actinic light at the irradiance phase 1) and F_0__i1′ (minimal Chl fluorescence under actinic light at the irradiance phase 1) were used to calculate operating efficiency of PSII (Φ_PSII_ = (${\text{F}}_{\text{m}}^{{\prime }}$ − ${\text{F}}_{0}^{{\prime }}$)/${\text{F}}_{\text{m}}^{{\prime }}$) and complete non-photochemical quenching (NPQ = (F_m_ − ${\text{F}}_{\text{m}}^{{\prime }}$)/${\text{F}}_{\text{m}}^{{\prime }}$).

**Additional file 3: Figure S3.** Calculation of the incoming flux generated by the Rh X-ray tube operating at 50 kV and 600 µA, in air, with primary emission filter Al 100 µm| Ti 25 µm, in the µXRF measurements. (a) Spectrum in linear scale, (b) Spectrum in log scale, (c) distribution by energy region.

**Additional file 4: Figure S4.** The effect of the multilayer mask on the detectors of the µXRF machine, to reduce spurious counts of Ni, Cu and Zn: (a) The spectrum for the blank, i.e. scatter on the plexiglass table. The mask eliminates almost all spurious counts from Zn and Cu, and most of Ni. (b) Spectrum of a pepper (*Capsicum annuum*) leaf measured with and without the mask, demonstrating that the mask only diminished the spurious counts but not the true signal from the sample.

**Additional file 5: Figure S5.** Assessing the space resolution of the µXRF measurements: scanning of the edge of a 30 µm thick Al foil with and without an Al–Ti primary emission filter.

**Additional file 6: Table S1.** Metal concentrations from ICP-MS for crop plants: pepper leaf (*C.* *annuum*, cultivar ‘Kozy Roh’) and soybean roots (*G.* *max*, cultivar ‘Galina’). Data for the pepper were collected from leaves of the same age and the same plants as the leaves used for the µXRF studies, though not the same leaves. Root data were obtained from a pooled, homogenized sample of four plants. The values represent averages ± SE.

## Data Availability

All data generated or analysed during this study are included in this published article and its additional files.

## Abbreviations

µXRF: Micro X-ray fluorescence
ddH2O: Double distilled water
EDX: Energy dispersive X-ray analysis
EDXMA: Energy dispersive X-ray microanalysis
FWHM: Full width at half maximum of the peak
HHNS: Hyperaccumulator hydroponic nutrient solution
ICP-MS: Inductively coupled plasma mass spectrometry
ICP-sfMS: Inductively coupled sector field plasma mass spectrometry
LA-ICP-MS: Laser ablation-inductively coupled plasma mass spectrometry
LOD: Limit of detection
OCR: Output count rate
OJIP: In vivo fluorescence rise from the initial state with open reaction centres (O) via reduction of plastoquinone A (J) and B (I) to reduction of the plastoquinone pool (P) [48]
PFA: Perfluoroalkoxy alkanes
PSI: Photosystem I
PSII: Photosystem II
PTFE: Polytetrafluoroethylene
RSE %: Relative standard error percentage
