# Supplementary material for: Analysis of trace metal distribution in plants with lab-based microscopic X-ray fluorescence imaging
Source: Plant Methods. 2020 Jun 8;16:82. doi: 10.1186/s13007-020-00621-5 (PMC7278123; doi:10.1186/s13007-020-00621-5)

Additional file 1: Fig. S1. Scheme of the sample mounting in the measuring chamber for both the µXRF and chlorophyll fluorescence kinetics measurements. The specimen is gently pressed against a printer foil window with a cotton pad (to avoid damage) and a 3-D printed polycarbonate plate to keep the specimen straight. A nylon mesh is used to press everything against the window by fixing it with an O-ring around the cover lid rim. The holes grid in the polycarbonate plate and the permeability of fine nylon mesh ensures the supply of air and humidity to the specimen.

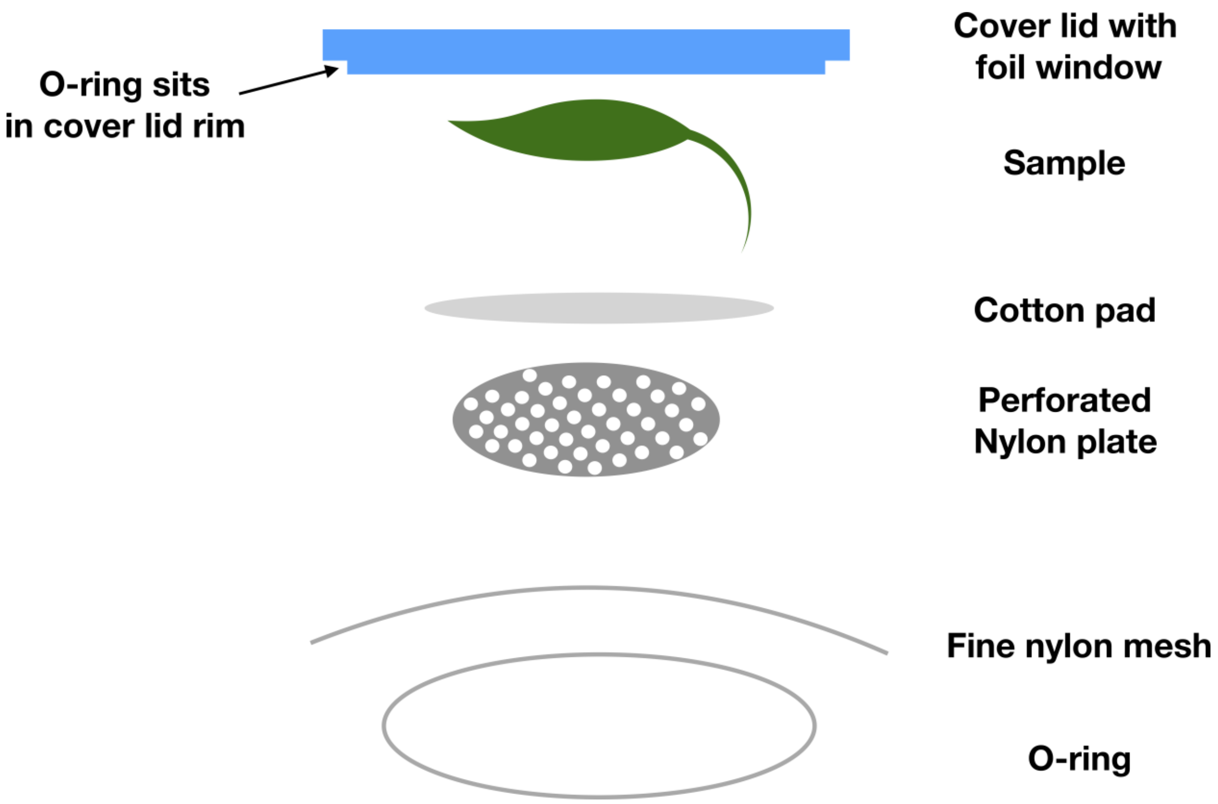

Supplement: Supplementary file 1 — Additional file 1: Figure S1. Scheme of the sample mounting in the measuring chamber for both the µXRF and chlorophyll fluorescence kinetics measurements. The specimen is gently pressed against a printer foil window with a cotton pad (to avoid damage) and a 3-D printed polycarbonate plate to keep the specimen straight. A nylon mesh is used to press everything against the window by fixing it with an O-ring around the cover lid rim. The holes grid in the polycarbonate plate and the permeability of fine nylon mesh ensures the supply of air and humidity to the specimen. [file 13007_2020_621_MOESM1_ESM.pdf]
