# Supplementary material for: Analysis of trace metal distribution in plants with lab-based microscopic X-ray fluorescence imaging
Source: Plant Methods. 2020 Jun 8;16:82. doi: 10.1186/s13007-020-00621-5 (PMC7278123; doi:10.1186/s13007-020-00621-5)

Additional file 2: Fig. S2. Chlorophyll fluorescence kinetic measurements of a pepper leaf. The intensity of the supersaturating flashes was 3,500 µmol m^{-2} s^{-1} and actinic light was 100 µmol m^{-2} s^{-1}. Intensities of F_0 (minimal Chl fluorescence of the dark-adapted leaf). Fm (maximal Chl fluorescence of the dark-adapted leaf), F_m_i1’ (maximal Chl fluorescence under actinic light at the irradiance phase 1) and F_0_i1’ (minimal Chl fluorescence under actinic light at the irradiance phase 1) were used to calculate operating efficiency of PSII (Φ_{PSII} = (F_m’-F_0’)/ F_m’) and complete non-photochemical quenching (NPQ=(F_m-F_m’)/F_m’).

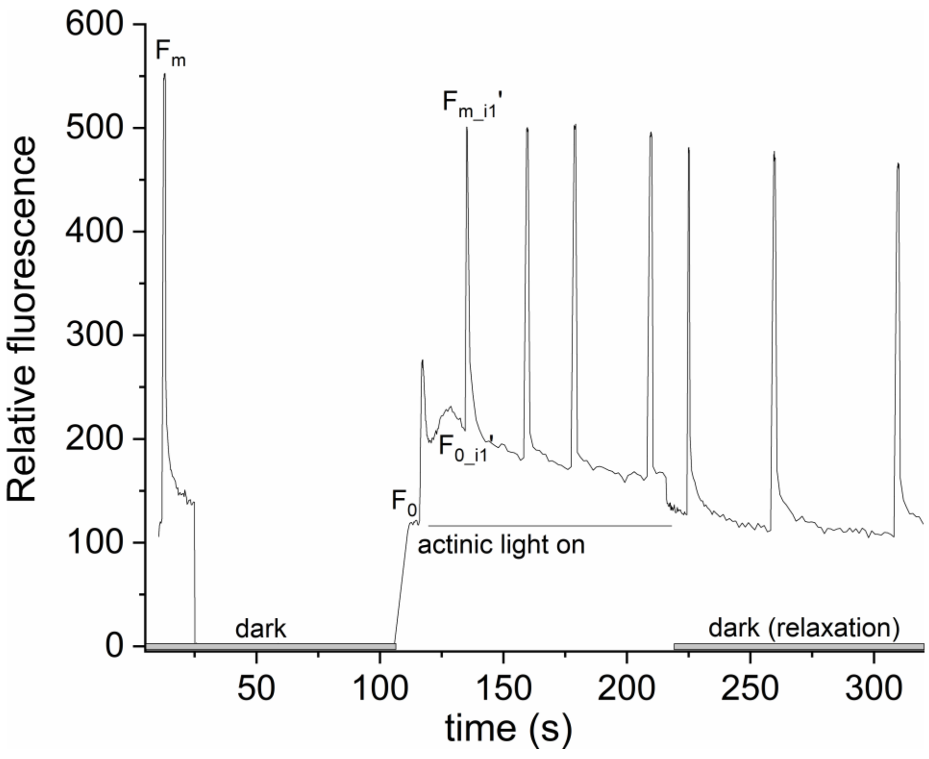

Supplement: Supplementary file 2 — Additional file 2: Figure S2. Chlorophyll fluorescence kinetic measurements of a pepper leaf. The intensity of the supersaturating flashes was 3500 μmol m−2 s−1 and actinic light was 100 μmol m−2 s−1. Intensities of F0 (minimal Chl fluorescence of the dark-adapted leaf). Fm (maximal Chl fluorescence of the dark-adapted leaf), Fm_i1′ (maximal Chl fluorescence under actinic light at the irradiance phase 1) and F0_i1′ (minimal Chl fluorescence under actinic light at the irradiance phase 1) were used to calculate operating efficiency of PSII (ΦPSII = (${\text{F}}_{\text{m}}^{{\prime }}$Fm′ − ${\text{F}}_{0}^{{\prime }}$F0′)/${\text{F}}_{\text{m}}^{{\prime }}$Fm′) and complete non-photochemical quenching (NPQ = (Fm − ${\text{F}}_{\text{m}}^{{\prime }}$Fm′)/ [file 13007_2020_621_MOESM2_ESM.pdf]
