# Supplementary material for: Analysis of trace metal distribution in plants with lab-based microscopic X-ray fluorescence imaging
Source: Plant Methods. 2020 Jun 8;16:82. doi: 10.1186/s13007-020-00621-5 (PMC7278123; doi:10.1186/s13007-020-00621-5)

Additional file 3: Fig. S3. Calculation of the incoming flux generated by the Rh X-ray tube operating at 50 kV and 600 µA, in air, with primary emission filter Al 100 µm | Ti 25 µm, in the µXRF measurements. a) Spectrum in linear scale, b) Spectrum in log scale, c) distribution by energy region.

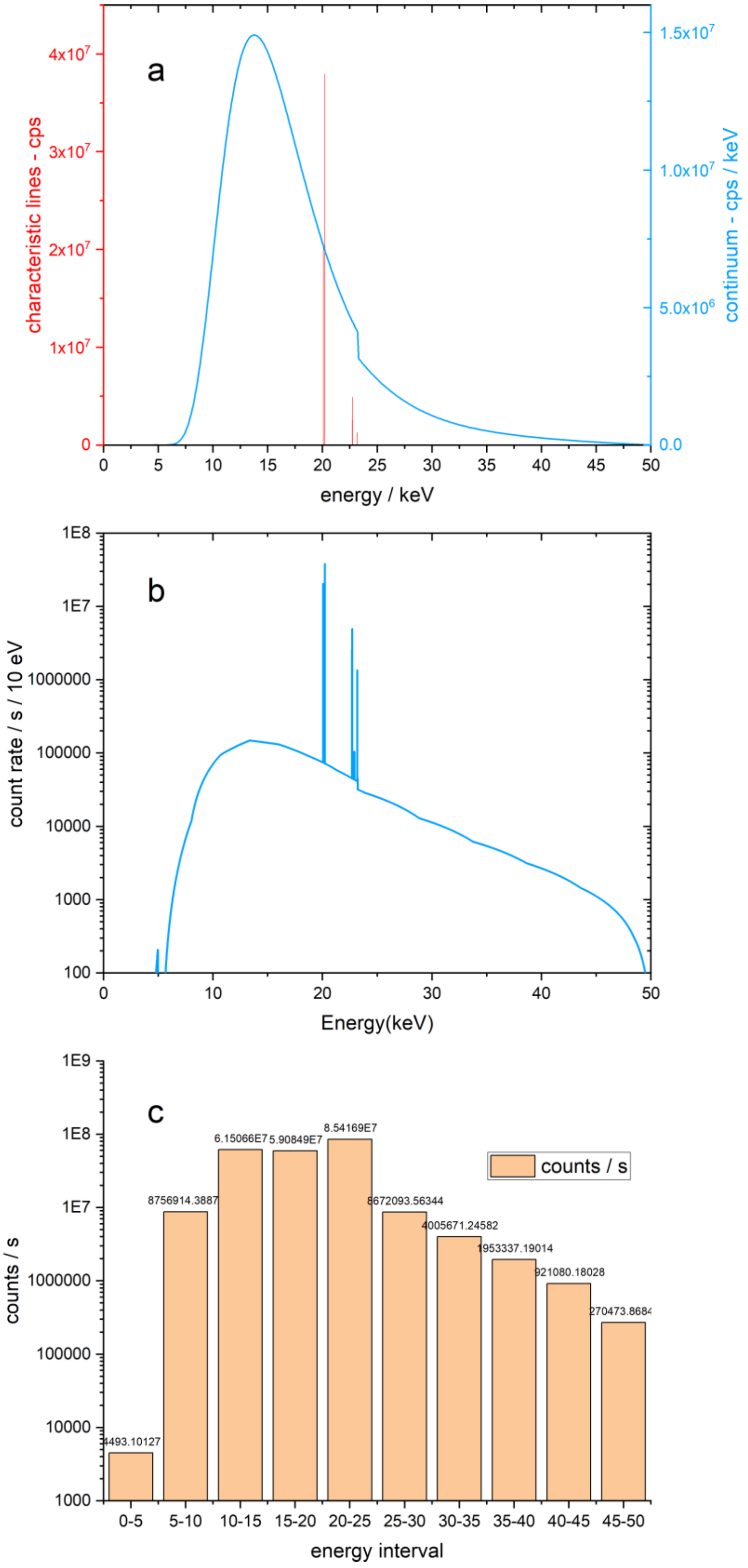

Supplement: Supplementary file 3 — Additional file 3: Figure S3. Calculation of the incoming flux generated by the Rh X-ray tube operating at 50 kV and 600 µA, in air, with primary emission filter Al 100 µm| Ti 25 µm, in the µXRF measurements. (a) Spectrum in linear scale, (b) Spectrum in log scale, (c) distribution by energy region. [file 13007_2020_621_MOESM3_ESM.pdf]
