# Supplementary material for: Analysis of trace metal distribution in plants with lab-based microscopic X-ray fluorescence imaging
Source: Plant Methods. 2020 Jun 8;16:82. doi: 10.1186/s13007-020-00621-5 (PMC7278123; doi:10.1186/s13007-020-00621-5)

Additional file 4: Fig. S4. The effect of the multilayer mask on the detectors of the µXRF machine, to reduce spurious counts of Ni, Cu and Zn: a) The spectrum for the blank, i.e. scatter on the plexiglass table. The mask eliminates almost all spurious counts from Zn and Cu, and most of Ni. b) Spectrum of a pepper (*Capsicum annuum*) leaf measured with and without the mask, demonstrating that the mask only diminished the spurious counts but not the true signal from the sample.

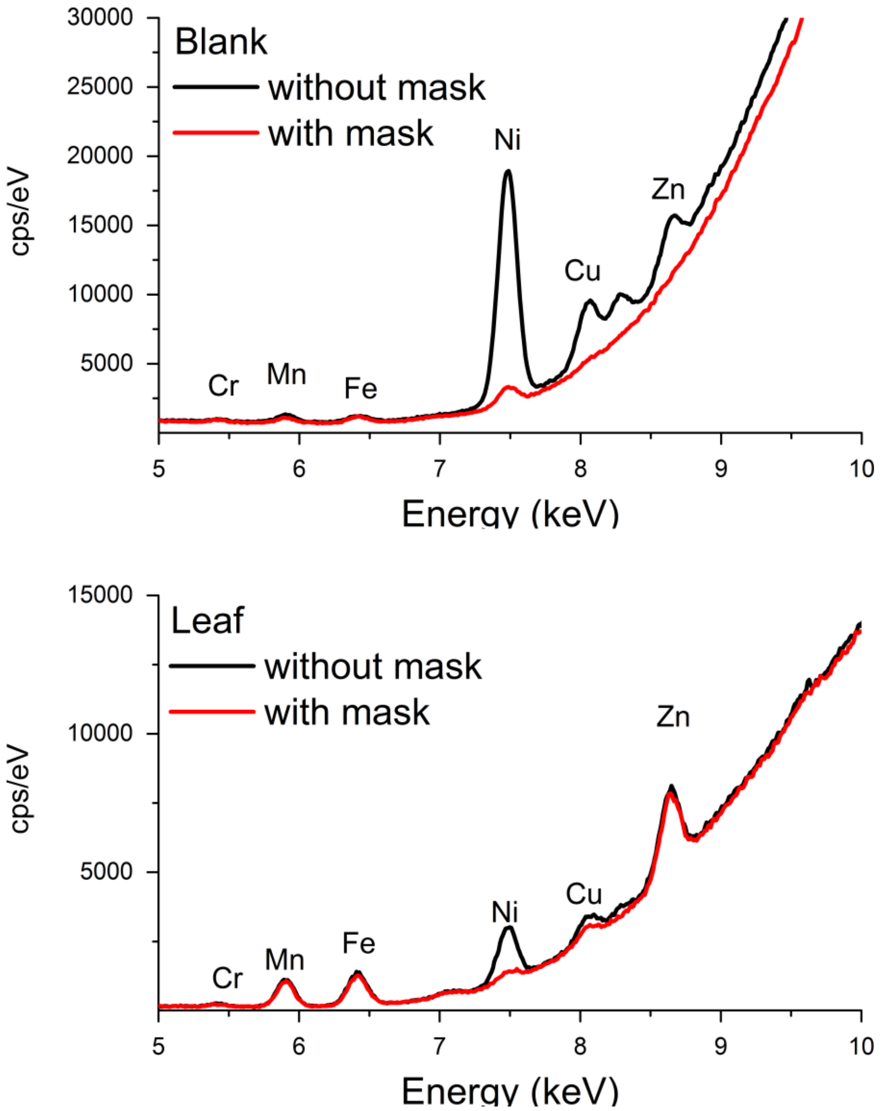

Supplement: Supplementary file 4 — Additional file 4: Figure S4. The effect of the multilayer mask on the detectors of the µXRF machine, to reduce spurious counts of Ni, Cu and Zn: (a) The spectrum for the blank, i.e. scatter on the plexiglass table. The mask eliminates almost all spurious counts from Zn and Cu, and most of Ni. (b) Spectrum of a pepper (Capsicum annuum) leaf measured with and without the mask, demonstrating that the mask only diminished the spurious counts but not the true signal from the sample. [file 13007_2020_621_MOESM4_ESM.pdf]
