# Supplementary material for: Analysis of trace metal distribution in plants with lab-based microscopic X-ray fluorescence imaging
Source: Plant Methods. 2020 Jun 8;16:82. doi: 10.1186/s13007-020-00621-5 (PMC7278123; doi:10.1186/s13007-020-00621-5)

Additional file 5: Fig. S5. Assessing the space resolution of the µXRF measurements: scanning of the edge of a 30 µm thick Al foil with and without an Al-Ti primary emission filter.

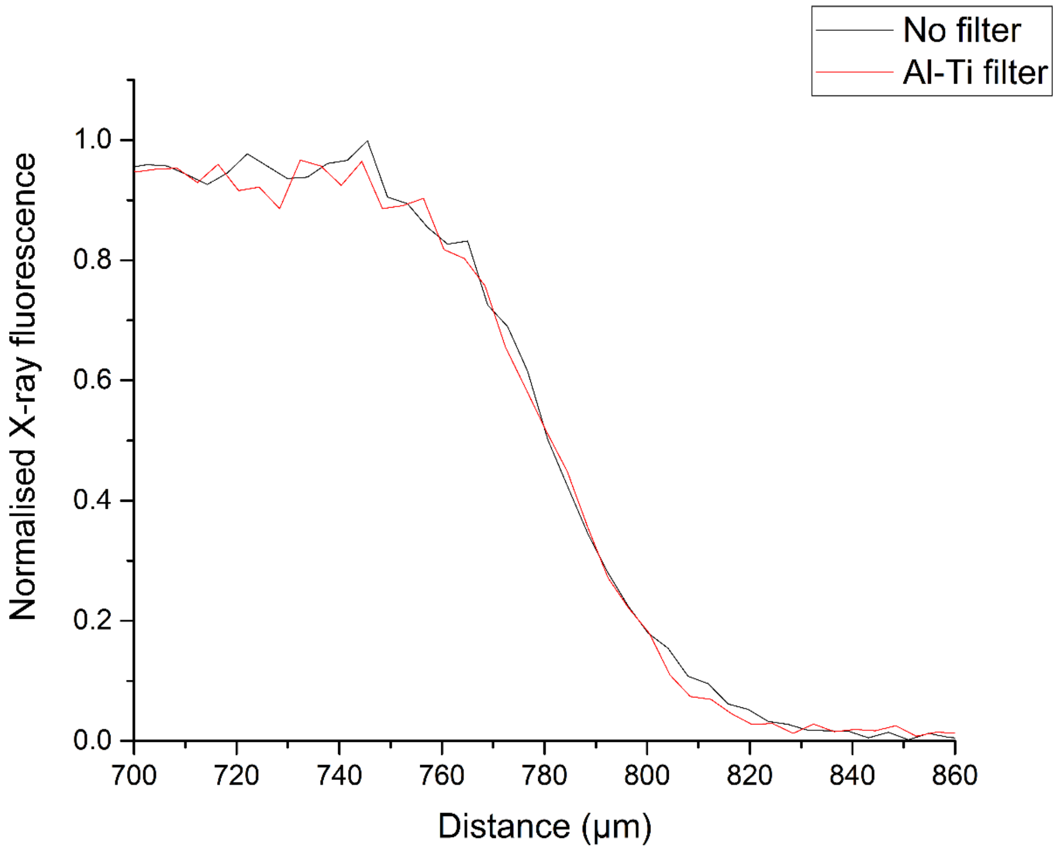

Supplement: Supplementary file 5 — Additional file 5: Figure S5. Assessing the space resolution of the µXRF measurements: scanning of the edge of a 30 µm thick Al foil with and without an Al–Ti primary emission filter. [file 13007_2020_621_MOESM5_ESM.pdf]
