# Supplementary material for: Analysis of trace metal distribution in plants with lab-based microscopic X-ray fluorescence imaging
Source: Plant Methods. 2020 Jun 8;16:82. doi: 10.1186/s13007-020-00621-5 (PMC7278123; doi:10.1186/s13007-020-00621-5)
Supplement: Supplementary file 6 — Additional file 6: Table S1. Metal concentrations from ICP-MS for crop plants: pepper leaf (C. annuum, cultivar ‘Kozy Roh’) and soybean roots (G. max, cultivar ‘Galina’). Data for the pepper were collected from leaves of the same age and the same plants as the leaves used for the µXRF studies, though not the same leaves. Root data were obtained from a pooled, homogenized sample of four plants. The values represent averages ± SE. [file 13007_2020_621_MOESM6_ESM.pdf]

Additional file 6: Table S1. Metal concentrations from ICP-MS for crop plants: pepper leaf (*C. annuum*, cultivar ‘Kozy Roh’) and soybean roots (*G. max*, cultivar ‘Galina’). Data for the pepper were collected from leaves of the same age and the same plants as the leaves used for the µXRF studies, though not the same leaves. Root data were obtained from a pooled, homogenized sample of four plants. The values represent averages ± SE.

Plant	Mn [mg kg^{-1}]	Fe [mg kg^{-1}]	Ni [mg kg^{-1}]	Cu[mg kg^{-1}]	Zn [mg kg^{-1}]
Pepper leaf	357 ± 16	155 ± 5	3.5 ± 0.1	12.0 ± 0.8	330 ± 27.
Soybean root	12 ± 5	29 ± 2	23 ± 5	6 ± 1	48 ± 10
